# Extracellular production of the engineered thermostable protease pernisine from *Aeropyrum pernix* K1 in *Streptomyces rimosus*

**DOI:** 10.1186/s12934-019-1245-3

**Published:** 2019-11-07

**Authors:** Marko Šnajder, Andrés Felipe Carrillo Rincón, Vasilka Magdevska, Miha Bahun, Luka Kranjc, Maja Paš, Polona Juntes, Hrvoje Petković, Nataša Poklar Ulrih

**Affiliations:** 1 Biotechnical Faculty, University of Ljubljana, Jamnikarjeva 101, 1000 Ljubljana, Slovenia; 2 Veterinary Faculty, University of Ljubljana, Ljubljana, Slovenia; 3 The Centre of Excellence for Integrated Approaches in Chemistry and Biology of Proteins, Ljubljana, Slovenia; 4 Present Address: AciesBio Ltd., Ljubljana, Slovenia; 5 Present Address: Labena Ltd., Ljubljana, Slovenia

**Keywords:** *Streptomyces rimosus*, Heterologous expression, Thermostable protease pernisine, *Aeropyrum pernix*, Prions

## Abstract

**Background:**

The thermostable serine protease pernisine originates from the hyperthermophilic *Archaeaon Aeropyrum pernix* and has valuable industrial applications. Due to its properties, *A. pernix* cannot be cultivated in standard industrial fermentation facilities. Furthermore, pernisine is a demanding target for heterologous expression in mesophilic heterologous hosts due to the relatively complex processing step involved in its activation.

**Results:**

We achieved production of active extracellular pernisine in a *Streptomyces rimosus* host through heterologous expression of the codon-optimised gene by applying step-by-step protein engineering approaches. To ensure secretion of fully active enzyme, the *srT* signal sequence from the *S. rimosus* protease was fused to pernisine. To promote correct processing and folding of pernisine, the *srT* functional cleavage site motif was fused directly to the core pernisine sequence, this way omitting the proregion. Comparative biochemical analysis of the wild-type and recombinant pernisine confirmed that the enzyme produced by *S. rimosus* retained all of the desired properties of native pernisine. Importantly, the recombinant pernisine also degraded cellular and infectious bovine prion proteins, which is one of the particular applications of this protease.

**Conclusion:**

Functional pernisine that retains all of the advantageous properties of the native enzyme from the thermophilic host was successfully produced in a *S. rimosus* heterologous host. Importantly, we achieved extracellular production of active pernisine, which significantly simplifies further downstream procedures and also omits the need for any pre-processing step for its activation. We demonstrate that *S. rimosus* can be used as an attractive host for industrial production of recombinant proteins that originate from thermophilic organisms.

## Background

The most common disadvantage of industrial enzymes is their loss of activity during storage or under extreme reaction conditions. Therefore, extremophilic organisms represent an attractive source of enzymes for industrial applications [1–3]. At present, the alkaline protease group of enzymes is the most frequently used in terms of industrial enzymes, with up to 30% of the global enzyme market share [4], with most of these used in the cleaning industry [5].

Pernisine is an alkaline protease from *Aeropyrum pernix* that belongs to the class of proteases designated as the subtilases, or more specifically, peptidase S8. It has been demonstrated that pernisine can efficiently degrade cellular and infectious bovine prion proteins [6], which today still present a challenge for decontamination processes, and this represents the main potential industrial application for pernisine. The protein sequence of fully unprocessed pernisine (i.e., prepropernisine) consists of 430 amino acids, which includes a putative signal sequence, as the first 24 amino acids, and a proposed proregion, from amino acids 25 to 92 [7, 8]. Pernisine is active at temperatures from 65 to 115 °C, and retains its catalytic activity across a wide pH range, from 4 to 10 [6].

The native host *A. pernix* produces pernisine at low yields, with an estimated final yield of around 0.5 mg/L culture medium [6]. At the same time, standard industrial fermentation facilities are not suitable for large-scale production of pernisine, due to its extreme cultivation conditions. Recombinant propernisine from *Escherichia coli* has similar properties to the wild-type enzyme, after an additional thermal activation step [7]. However, since the recombinant enzyme is located in the intracellular region or in the periplasm, additional downstream operations are required for its isolation and effective temperature pre-treatment. These isolation procedures are very difficult to scale up to the industrial volume while still ensuring sufficient quantities of fully active pernisine.

To overcome the limitations of the *E. coli* host, we applied a new expression system based on *Streptomyces rimosus*, which is known for efficient secretion of proteins [9]. *Streptomyces* spp. have two main secretory pathways: the Sec-dependent and the Tat-dependent pathways [10, 11]. Different signal peptides can lead to extracellular preproteins, where they are cleaved by signal peptidases [12, 13]. The main difference between these two secretion pathways is that for the Tat pathway, proteins fold into their final conformation inside the cell before they are secreted, whereas for the Sec pathway, proteins are folded after their secretion through the cell membrane [12, 13].

Although among the *Streptomyces* spp. *S. lividans* is the most frequently used host for production of heterologous proteins, it was recently demonstrated that *S. rimosus* is a further promising host [9]. Indeed, numerous industrially useful molecular and microbiology methods have been developed over decades of industrial development for the production of oxytetracycline with *S. rimosus* [11, 14]. Further, industrial fermentation procedures and the development of gene tool kits for extracellular production of heterologous proteins in *S. rimosus* have been described recently [9].

In the present study, we demonstrated the production in *S. rimosus* of codon-optimised fully processed and proteolytically active recombinant pernisine, with yields comparable to those for *E. coli* [7]. By applying synthetic-biology approaches, we constructed a functional fusion of the *S. rimosus* protease *srT* signal sequence [9] directly with the codon-optimised core *pernisine* gene (*pernisine*^*CO*^), thus omitting the prepro-region of the wild-type *pernisine* gene (*pernisine*^*WT*^). Efficient export of proteolytically active pernisine was achieved without the need for temperature pre-treatment, which significantly simplifies the downstream processing. Finally, we isolated the codon-optimised proteolytically active pernisine from the *S. rimosus* culture supernatant, which showed comparable catalytic properties to the wild-type enzyme and degradation of the infectious prion protein PrP^Sc^ from bovine brain homogenates. We have thus demonstrated that although challenging, it is possible to use *S. rimosus* as an alternative industrial host for production of functional enzymes from a thermophile.

## Results

**Fig. 1:**
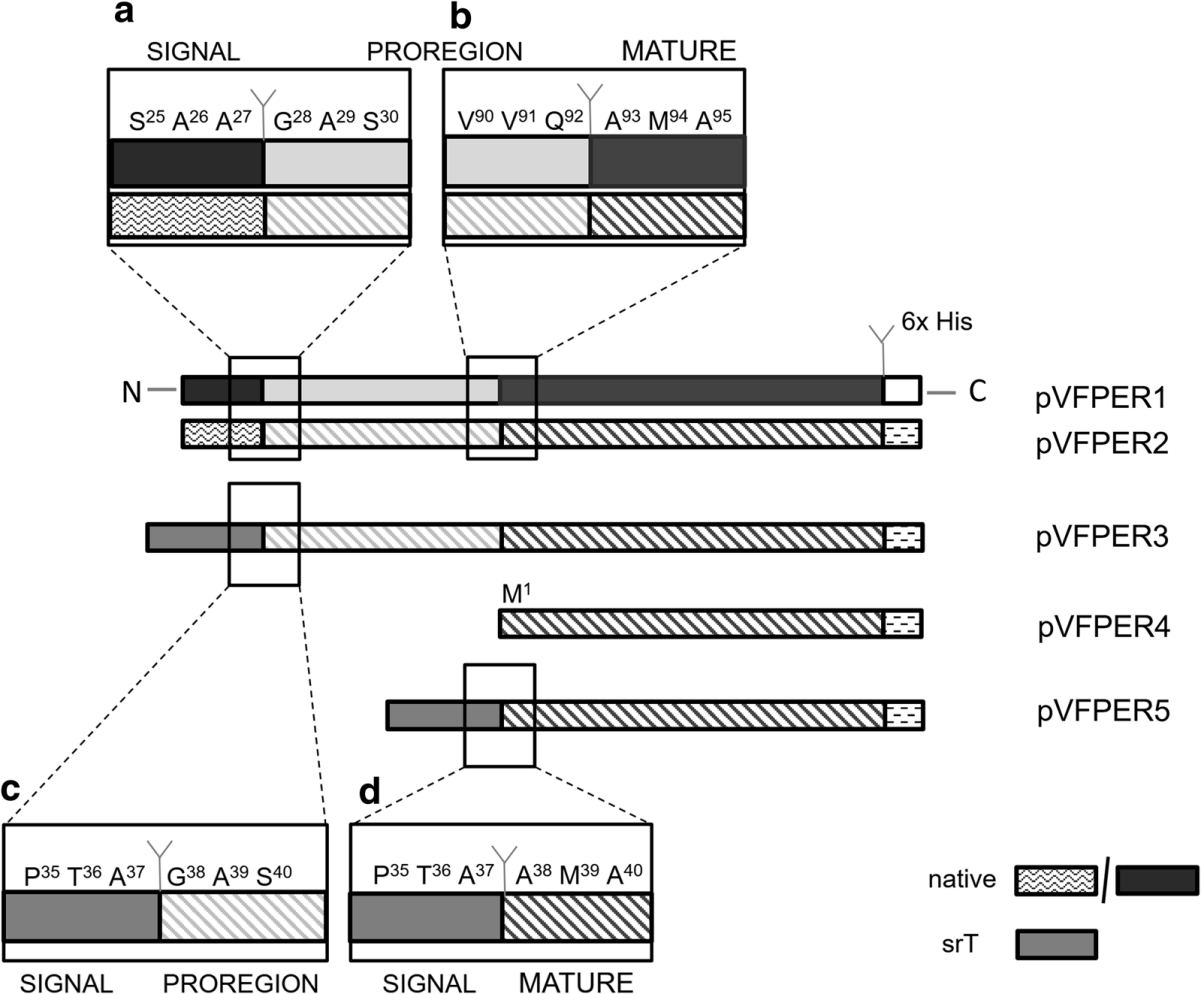
Schematic representation of the engineered *pernisine* genes subcloned into the pVF vector. The five plasmid constructs (pVFPER1-5) contained the *pernisine* mature domain and the C-terminal His_6_-tag. Plasmid pVFPER1 contained the preproregion with the wild-type signal sequence from *A. pernix* (*prepropernisine*^*WT*^). The other plasmids (pVFPER2-5) were codon-optimised, as: pVFPER2, *prepropernisine*^*CO*^, with the proregion and the wild-type signal sequence from *A. pernix*; pVFPER3, *propernisine*^*CO*^, with the wild-type signal sequence replaced by the *srT* signal sequence from *S. rimosus*; pVFPER4, with only the mature domain (*pernisine*^*CO*^); and pVFPER5, with the mature domain (*pernisine*^*CO*^) and the *srT* signal sequence from *S. rimosus* (i.e., fused directly to the core sequence of *pernisine*^*CO*^). The enlarged boxes show the predicted signal peptidase cleavage sites between the wild-type signal sequence and the proregion of *propernisine*^*WT/CO*^ (**a**), the proregion and the mature protein (*pernisine*^*WT/CO*^) (**b**), the *S. rimosus srT* signal sequence and the proregion (*propernisine*^*CO*^) (**c**), and the *srT* signal sequence and the mature protein (*pernisine*^*CO*^) (**d**). The Y-shaped lines show the predicted junctions between the different functional domains. The numbering of the positions of the selected amino acids is given

As described in the Materials and Methods, all of the versions containing the *pernisine* gene were cloned into the *E. coli*–*Streptomyces* replicative vector pVF, which contained the pIJ101 replicon. The target genes were expressed under the relatively strong P_*tcp830*_ promoter, which supports constitutive expression in the *S. rimosus* background, as described by [9]. Additionally, to simplify detection of the pernisine protein, sequence coding for six histidine amino-acid residues was fused to the C-terminal part of all of the gene constructs (Fig. 1). Thus, Western blotting and further isolation and protein characterisation was simplified.

We initially evaluated the possible production of a wild-type version of pernisine in the *S. rimosus* background through the construction of expression plasmids that contained the DNA sequences for the native pernisine (*prepropernisine*^*WT*^; Fig. 1, pVFPER1) and the native codon-optimised pernisine (*prepropernisine*^*CO*^; Fig. 1, pVFPER2) (Table 1).

**Table 1:** Expression vectors used in this study

Vector	Protein assembly	Theoretical MW (kDa)	Proregion	*S. rimosus* signal sequence	Codon optimized
pVFPER1	Prepropernisine-His_6_-C	43	Yes	No	No
pVFPER2	Prepropernisine-His_6_-C	43	Yes	No	Yes
pVFPER3	srT-propernisine-His_6_-C	45	Yes	Yes	Yes
pVFPER4	Pernisine-His_6_-C	36	No	No	Yes
pVFPER5	srT-pernisine-His_6_-C	38	No	Yes	Yes

### Engineering of the native and codon-optimised *prepropernisine* gene and analysis of protein production

The wild-type pernisine gene, *prepropernisine*^*WT*^, was PCR amplified with specific primers (see Materials and Methods) using genomic DNA of *A. pernix* as the template, which was then subcloned into the pVF vector to generate pVFPER1, as shown in Fig. 1 and Table 1. This was initially tested for expression of *prepropernisine*^*WT*^ in *S. rimosus*. Although 20 independent *S. rimosus* transformants that contained the pVFPER1 plasmid were tested, we did not detect pernisine, independent of whether its intracellular or extracellular activity was evaluated, or whether SDS-PAGE, Western blotting or zymography assays were used.

**Fig. 2:**
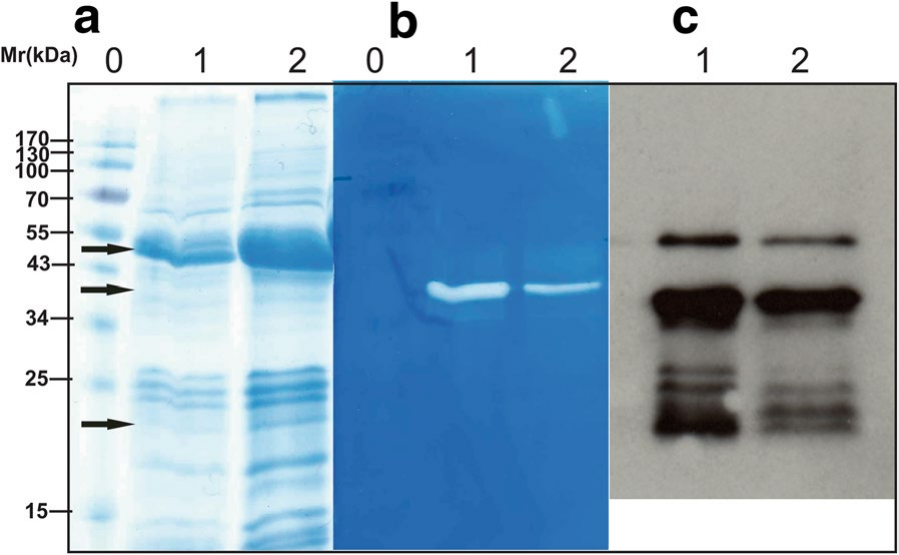
Analysis using SDS-PAGE (**a**), zymography (**b**) and Western blotting (**c**) following the initial trial for activation of *prepropernisine*^*CO*^ from total cell lysates of *S. rimosus* transformed with plasmid construct pVFPER2. Lane 0, molecular weight markers; Lane 1, prepropernisine in 10 mM HEPES, pH 8.0, 1 mM CaCl_2_; Lane 2, heat-activated prepropernisine (30 min, 80 °C). Processed prepropernisine bands are indicated with arrows

Furthermore, we tested 20 independent *S. rimosus* transformants that contained the plasmid construct pVFPER2 with codon-optimised prepropernisine (*prepropernisine*^*CO*^). Numerous bands appeared on the SDS-PAGE gels (Fig. 2a). The zymography assay showed limited activity of the isolated *prepropernisine*^*CO*^ seen as the 36 kDa band in Fig. 2b. When Western blotting was performed, additional bands appeared at 52 kDa and 23 kDa (Fig. 2c). The 52-kDa band was assumed to be the unprocessed prepropernisine and the 23-kDa band to be the autodegradation product of pernisine (Fig. 2c).

Tandem mass spectrometry (MS/MS) analysis of the protein at 52 kDa confirmed that it was prepropernisine (Fig. 2, Additional file 1: Fig. S1A). It was not possible to confirm the composition of the 36-kDa band, which appeared to contain only a proteolytically active fraction (Fig. 2b), due to the low yields of the isolated protein. Thus, we can conclude that the codon-optimised *prepropernisine*^*CO*^ was successfully expressed in *S. rimosus*. However, the lack of processing of the prepropernisine led to the consideration of new approaches for the production of active pernisine.

It is important to emphasise at this point that all of the engineered *pernisine* genes constructed further on were based on the codon-optimised *prepropernisine*^*CO*^ gene, namely, pVFPER3, pVFPER4 and pVFPER5 (Fig. 1, Table 1).

### Engineering of the pernisine core region of *prepropernisine*^*CO*^ fused directly to the signal sequence *srT*

For the design and construction of the pernisine constructs that contained pernisine with (*propernisine*^*CO*^) and without (*pernisine*^*CO*^) the proregion, we applied the model where the native signal peptide is predicted to occur from M^1^ to A^27^ (Fig. 1a, b), and the proregion from G^28^ to Q^92^ (Additional file 1: Fig. S2A). The detailed results of the bioinformatics analysis are described in Additional file 1: Supplemental results.

### Construction of different *propernisine*^*CO*^ versions that contain the *S. rimosus* protease *srT* signal sequence

Three different plasmid constructs were prepared. First, the *propernisine*^CO^ gene was constructed where the wild-type signal sequence of *prepropernisine*^*CO*^ was removed and replaced by the *srT* signal sequence, to generate pVFPER3. The fusion site between the wild-type signal sequence and *propernisine* was determined using the Pred-TAT software, which identified a cleavage site of the archaeal signal sequence at A^27^-G^28^. To conserve the A–X–A motif required for signal peptidase cleavage [15], the *srT* signal sequence was fused to *propernisine*^*CO*^ in a manner that conserved the recognition motif (Fig. 1c). It should be noted that the *srT* signal peptide is 10 amino acids longer than the native signal peptide. The ‘synthetic’ in silico constructed cleavage site was analysed again using the Pred-TAT software, which showed that the predicted cleavage site between the *srT* signal sequence and *propernisine*^*CO*^ was at A^37^-G^38^, which confirmed that the cleavage site did not change, and it was used to construct the pVFPER3 plasmid (Fig. 1c). Therefore, the pVFPER3 construct still contained the proregion, which was fused to the *S. rimosus* signal sequence *srT*.

In the second approach, with the aim to avoid the propernisine processing step, the *P*_*tcp830*_ promoter was fused directly to *pernisine*^*CO*^, which resulted in construction of the pVFPER4 plasmid (Fig. 1). In this case, the signal sequence *srT* for export of the protein out of *S. rimosus* was not present. Therefore, although toxicity to the *S. rimosus* host might occur in this case, the aim was for fully processed pernisine to be produced intracellularly. Based on alignment with the Tk-subtilisin amino-acid sequence and its three-dimensional (3D) structure [8] as described above, we defined the proregion cleavage site of propernisine as Q^92^-A^93^ (Fig. 1b). Based on these data, *pernisine*^*CO*^ was amplified by PCR. The NdeI restriction site was then engineered into the design of the forward primer, thus incorporating the start codon (M^1^) and the restriction site for pVFPER4 vector construction. To detect the production of pernisine intracellularly, initial *S. rimosus* cell lysis had to be performed, as described in the Materials and Methods.

Finally, in the third approach, to avoid the pernisine activation step and simultaneously achieve secretion of proteolytically active pernisine into the culture medium, we constructed a modified *pernisine*^*CO*^ that lacked both the wild-type signal sequence and the proregion, which thus lacked the first 92 amino acids of prepropernisine. Here, the *srT* signal sequence for secretion was added directly to the core *pernisine* region, which provided plasmid construct pVFPER5 (Fig. 1, Table 1). This approach combined the construction strategies of the pVFPER3 and pVFPER4 plasmids. The *srT* signal sequence was fused to *pernisine*^*CO*^, which resulted in A^37^-A^38^ (Fig. 1d), where the conserved the A–X–A signal peptidase recognition motif was also confirmed with the Pred-TAT software. We thus assayed for the direct production of pernisine that might be exported directly out of the cell. These plasmid constructs are described in more detail in Table 1 and Fig. 1.

All three constructs were transformed into *S. rimosus* by electroporation, and 20 independent transformants that contained each plasmid (i.e., pVFPER 3, 4, 5) were analysed by SDS-PAGE and zymography, as described in the Materials and methods.

**Fig. 3:**
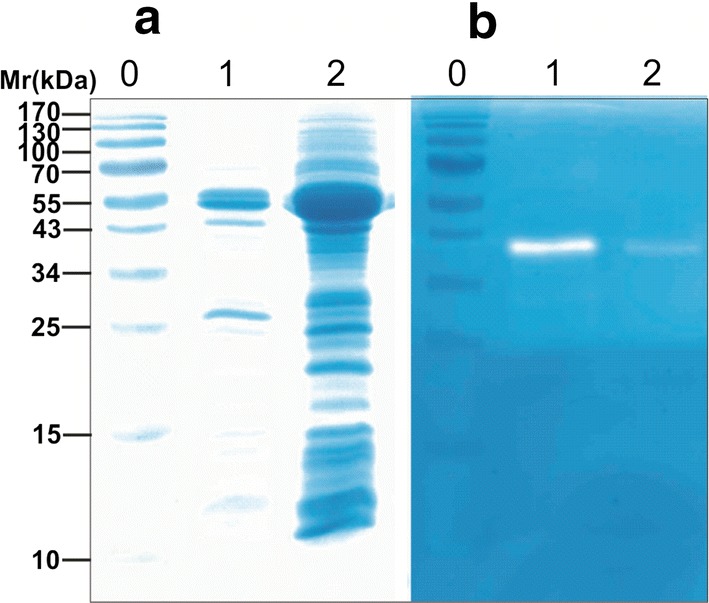
Codon optimised srT-propernisine (plasmid construct pVFPER3) in the intracellular and extracellular cell fractions following purification using affinity chromatography and analysis by SDS-PAGE (**a**) and zymography (**b**). Lanes 0, protein molecular weight markers; lanes 1, intracellular codon-optimised srT-pernisine; lanes 2, extracellular codon-optimised srT-pernisine

Three selected *S. rimosus* transformants with the plasmid construct pVFPER3 that contained *srT*-*propernisine*^*CO*^ were cultivated, and their intracellular fraction and culture supernatant were analysed with SDS-PAGE and zymography assays. Unprocessed propernisine was identified in the culture medium (Fig. 3), which confirmed that srT-propernisine^CO^ was expressed and that the propernisine was exported into the culture medium. This also confirmed the functionality of the *srT* signal sequence. However, the processing of propernisine to produce fully active pernisine did not occur (Fig. 3b).

**Fig. 4:**
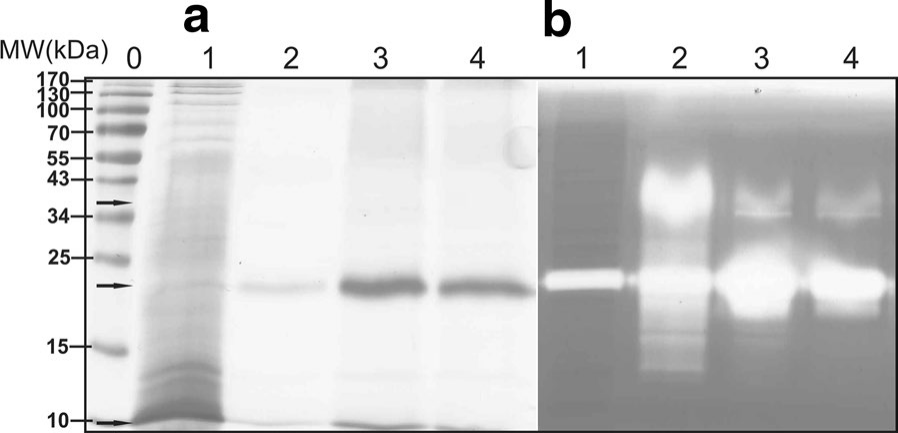
Analysis of the isolated and purified codon-optimised pernisine produced by *S. rimosus* transformants transformed with plasmid construct pVFPER5, using SDS-PAGE (**a**) and zymography (**b**). Purified codon-optimised pernisine was visualised on standard 15% SDS-PAGE (**a**) and 15% SDS-PAGE with casein as substrate (**b**) for the zymography activity (2 h at 80 °C). Lane 0, molecular weight markers (indicated left); Lane 1, cell lysate fraction; Lane 2, fraction from Ni–NTA chromatography; Lane 3, fraction from preparative grade gel filtration column (Superdex 200); Lane 4, dialysed codon-optimised pernisine fraction

**Fig. 5:**
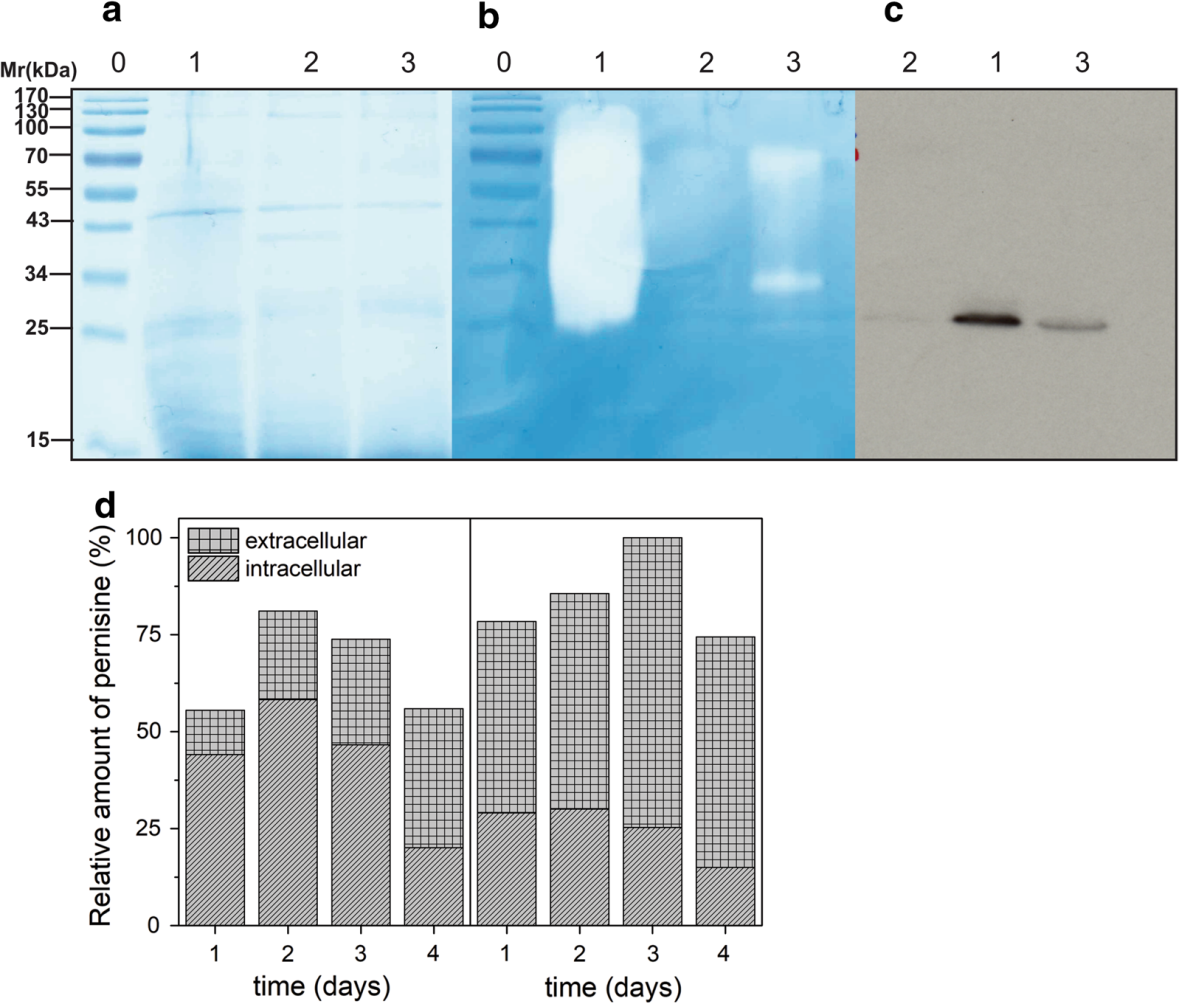
Analysis of codon-optimised pernisine (plasmid construct pVFPER4) and srT-pernisine (plasmid construct pVFPER5) from culture supernatants from *S. rimosus*. Proteins from culture supernatant were separated by SDS-PAGE (**a**) and zymography (**b**). The proteins were transferred onto nitrocellulose membranes and His_6_-tagged pernisine was detected using anti-His_6_ tag antibodies (**c**). Lanes 0, protein MW markers; lanes 1, srT-pernisine^CO^; lanes 2, negative control; lanes 3, pernisine^CO^. **d** Additionally, the amounts of secreted pernisine^CO^ (left) and srT-pernisine^CO^ (right) were estimated using dot blot assays

In contrast, when culturing *S. rimosus* transformants that contained *pernisine*^*CO*^ and *srT*-*pernisine*^*CO*^ encoded in pVFPER4 and pVFPER5, respectively, we observed the processed and proteolytically active pernisine at 23 kDa by SDS-PAGE (Figs. 4a, 5a). Zymography and Western blotting also confirmed this proteolytically active 23-kDa protein (Fig. 5b, c), and MS/MS analysis confirmed that the isolated protein was indeed pernisine, which comprised amino acids 137-430 (Additional file 1: Fig. S1B). In addition to the 23-kDa band on SDS-PAGE, a band of 60 kDa was detected (Fig. 5b, lane 3), which appeared to be an oligomer of pernisine. The removal of the proregion and the wild-type signal sequence from the core pernisine gene and its fusion with the *srT* signal sequence from *S. rimosus* (to generate *srT*-*pernisine*^*CO*^, pVFPER5) resulted in up to three-fold higher secretion of codon-optimised and fully processed recombinant pernisine, as estimated from Western blotting (Fig. 5c) and dot blots (Fig. 5d).

Considering that fully processed pernisine with the highest yield was obtained by applying *srT*-*pernisine*^*CO*^ (pVFPER5, Fig. 1), all of the further pernisine characterisation was carried out using *S. rimosus* transformants containing pVFPER5, unless otherwise specified.

### Selection of production medium

To increase the yield from the recombinant *srT*-*pernisine*, several different media were evaluated for cultivation of the *S. rimosus* transformants, with monitoring using dot-blot and zymography assays. The highest signal was observed in cultivations with complex medium, 3 days after inoculation (Fig. 5d, Additional file 1: Fig. S3). Detailed evaluation of the different media is described in Additional file 1: Supplemental results.

### Isolation of codon-optimised recombinant pernisine and evaluation of its biochemical properties

Considering that *srT*-*pernisine*^*CO*^ (pVFPER5) expressed in *S. rimosus* produced the highest yield of extracellular pernisine when cultivated in complex medium, this procedure was used for the subsequent production and isolation of pernisine. The recombinant pernisine was isolated using Ni–NTA affinity chromatography. When high purity was needed (> 95%), size-exclusion chromatography was added as a purification step, as described previously [7]. This provided pernisine with > 90% purity, as estimated by SDS-PAGE (Fig. 4a), which was used for the biochemical characterisation.

### Biochemical characterisation of the recombinant pernisine

**Fig. 6:**
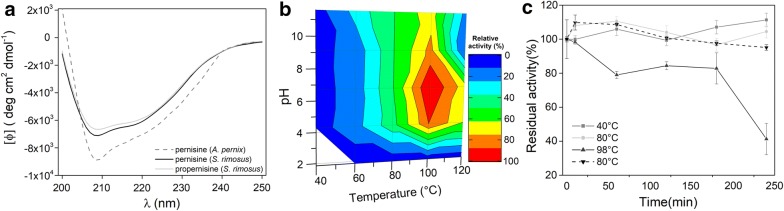
**a** Far-UV circular dichroism spectra of the codon-optimised recombinant propernisine (black line; plasmid construct pVFPER3) and pernisine (grey line; plasmid construct pVFPER5) from *S. rimosus*, and pernisine from *A. pernix* (dashed line). The spectra of the purified proteins (0.2 mg/mL) were measured at pH 8.0 (10 mM Tris/HCl) at 25 °C. **b** Simultaneous influence of temperature and pH on the activity of the codon-optimised recombinant pernisine (plasmid construct pVFPER5). Data are means for the relative proteolytic activities from experiments carried out in triplicate, as a function of temperature and corrected for pH. Colour bars: relative activities. **c** Time-courses for the temperature stabilities (as indicated) of the proteolytic activities for codon-optimised purified recombinant pernisine (from plasmid construct pVFPER5) produced by *S. rimosus* (black squares, grey circles, dark grey triangles) and wild-type pernisine (dashed line, inverted triangles)

To identify the amino acids of pernisine that act as Ca^2+^ binding sites, a 3D structural model was built based on the known 3D structure of Tk-subtilisin [16] (Additional file 1: Fig. S2). Amino-acid alignment of pernisine with Tk-subtilisin showed that > 80% of the amino acids were identical or very similar (Additional file 1: Fig. S4). For pernisine, the model structure of the proteolytically active pernisine (Additional file 1: Fig. S2) identified seven potential Ca^2+^ binding sites, which is analogous to that of Tk-subtilisin (Additional file 1: Figs. S2C, S4). The secondary structure analysis here using circular dichroism (CD) revealed that the 52-kDa form of the recombinant prepropernisine had 21.0% ± 0.1% α-helices and 33.0% ± 0.2% β-sheets, whereas the fully processed and proteolytically active 23-kDa form of pernisine had 22.0% ± 0.2% α-helices and 27.0% ± 0.2% β-sheets (Fig. 6a). For comparison, the CD spectra of proteolytically active wild-type pernisine from *A. pernix* showed 27.0% ± 0.2% α-helices and 21.0% ± 0.3% β-sheets (Fig. 6a).

The CD data (Fig. 6a) plus the zymography (Fig. 4b) showed that the proregion of pernisine is not essential for its correct folding. The CD spectra (Fig. 6a) and the estimated secondary structural elements of prepropernisine and pernisine showed very small differences when the proregion was omitted: a 1% increase in α-helices, and a 6% decrease in β-sheets.

### Effects of pH and temperature on proteolytic activity

The proteolytic activities of the codon-optimised recombinant pernisine from *S. rimosus* were determined at different temperatures (40, 80, 98 °C), at pH 7.9 (± 0.2), and over different incubation times (0, 10, 60, 120, 180, 240 min). These proteolytic activities of the recombinant and wild-type pernisine remained unchanged after 4 h at 80 °C (Fig. 6c). After 2 h at 98 °C, the recombinant pernisine retained ~ 80% of its proteolytic activity; however, an additional 1 h at 98 °C reduced this to 30%. Based on this and previous studies, we can conclude that the codon-optimised recombinant pernisine is as stable as wild-type pernisine [6]. To investigate the catalytic properties of the codon-optimised recombinant pernisine, an azocasein assay was performed for proteolytic activity, from pH 1.9 to 11.6, and from 40 °C to 120 °C. As shown in Fig. 6b, the 3D representation of the simultaneous effects of temperature and pH on the relative proteolytic activity of the codon-optimised recombinant pernisine from *S. rimosus* showed > 90% relative activity from pH 4.5 to 7.0 in the temperature range from 95 to 105 °C.

### Effect of inhibitors, denaturing agents and detergent on proteolytic activity

The residual proteolytic activities of the codon-optimised recombinant pernisine isolated from *S. rimosus* cultures were determined under treatments with different protease inhibitors and denaturing agents using azocasein assays (see “Materials and methods” and Additional file 1: Materials and Methods). Further evaluation and comparisons between wild-type pernisine and this recombinant pernisine from *S. rimosus* were performed, following the previous investigations on recombinant pernisine from *E. coli* [6, 7, 17]. This comparison included the three variants of pernisine, as wild-type, a recombinant pernisine from *E. coli*, and the recombinant pernisine from *S. rimosus* (Tables 2, 3).

**Table 2:** Residual protease activities of recombinant pernisine in the presence of the protease inhibitors

Addition	Concentration	Residual proteinase activity at 1 mM CaCl_2_ (%)		
		Present study	Recombinant pernisine in *E. coli*^a^	Native pernisine^b^
None	–	100.0 ± 5.3	100.0 ± 1.7	100.0
EDTA	1	78.6 ± 6.7	92.8 ± 0.6	93.5
	5	0.5 ± 4.7	2.1 ± 0.1	0.5
EGTA	1	85.7 ± 5.4	90.5 ± 5.5	91.2
	5	6.0 ± 1.0	8.2 ± 1.0	1.4
PMSF	1	39.5 ± 4.4	6.1 ± 0.8	6.9
	10	21.0 ± 3.4	0.9 ± 0.5	2.8
IAA	1	111.1 ± 8.2	69.1 ± 6.5	ND
	10	99.6 ± 5.4	89.6 ± 2.0	91.7

*ND* no data, *EDTA* ethylenediaminetetraacetic acid, *EGTA* ethylene glycol-bis(b-aminoethyl ether)-*N,N,N,N*-tetraacetic acid, *PMSF* phenylmethylsulphonyl fluoride, *IAA* iodoacetamide

^a^Šnajder et al. [7]

^b^Šnajder et al. [6]

**Table 3:** Residual protease activity of recombinant pernisine in the presence of reducing agents, denaturants, and a detergent

Reagent	Concentration	Residual proteinase activity at 1 mM CaCl_2_ (%)		
		Present study	Recombinant pernisine in *E. coli*^a^	Native pernisine^b^
None	–	100.0 ± 5.3	100 ± 4.7	100.0
DTT	1 mM	79.3 ± 4.0	58.0 ± 2.3	27.9
	5 mM	63.7 ± 1.8	47.9 ± 4.5	25.9
2-MeEtOH	1%	78.5 ± 7.6	50.6 ± 11.6	34.8
	5%	63.1 ± 2.6	38.4 ± 2.5	30.8
Gdn-HCl	1 M	218.6 ± 24.7	124.9 ± 4.0	66.2
	4 M	189.0 ± 12.3	189.6 ± 10.1	152.2
Urea	1 M	136.0 ± 9.0	119.7 ± 9.7	51.7
	4 M	121.8 ± 9.7	106.0 ± 3.1	46.8
SDS	0.10%	107.0 ± 8.1	90.8 ± 0.8	65.7
	3%	22.3 ± 3.3	10.4 ± 2.8	33.8

*DTT* dithiothreitol, *2-MeEtOH* 2-mercaptoethanol, *Gdn-HCl* guanidinium hydrochloride, *SDS* sodium dodecil sulphate

^a^Šnajder et al. [7]

^b^Šnajder et al. [6]

Interestingly, in the presence of the serine protease inhibitor phenylmethylsulfonyl fluoride (10 mM), the recombinant fully processed pernisine from *S. rimosus* still retained 21.0% of its residual proteolytic activity, compared to the recombinant fully processed pernisine from *E. coli* at 0.9%, and the wild-type pernisine at 2.8% (Table 2). This recombinant protein from *S. rimosus* showed higher resistance to the effects of reducing agents, denaturants and a detergent, and thus higher residual proteolytic activities of 10% to 30% (Table 3). This indicates the advantages of this recombinant pernisine from *S. rimosus* compared to pernisine produced in *E. coli*. Independent of the source, similar residual proteolytic activities of pernisine were seen in the presence of the chelating agents EDTA and EGTA (Table 2).

### Degradation of the prion protein in bovine-brain homogenates

**Fig. 7:**
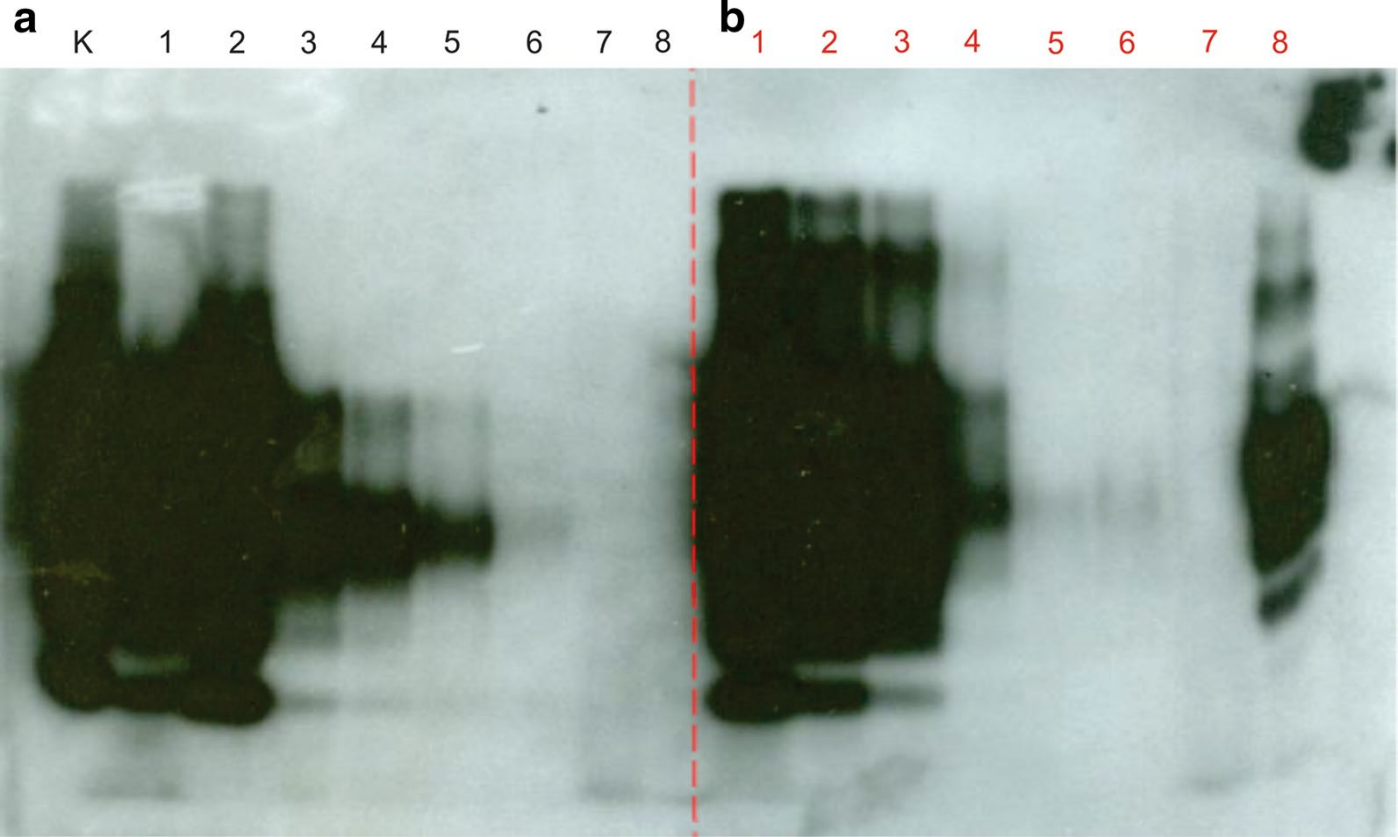
Western blotting with Prionics–Check Western kit (PCW) showing degradation of bovine-brain homogenate containing normal cellular prion protein (PrP^c^) (**a**, lanes 1–6) and infectious prion protein (PrP^Sc^) (**b**, lanes 1–6) by the codon-optimised recombinant pernisine (from plasmid construct pVFPER5) from *S. rimosus* (lanes 1–6), and at different dilutions of the brain homogenates in the reaction mixtures. PCW brain homogenate, undiluted (lane 1), and PCW brain homogenate diluted with water (v/v) 1:1 (lane 2), 1:2 (lane 3), 1:3 (lane 4), 1:4 (lane 5) and 1:5 (lane 6). Controls to the codon-optimised recombinant pernisine: untreated control (**a**, lane K); wild-type pernisine (**a**, **b**, lane 7; brain homogenate dilution 1:1); proteinase K from the kit (**a**, **b**, lane 8; brain homogenate dilution 1:1)

Considering the potential commercial use of recombinant pernisine from *S. rimosus,* this codon-optimised recombinant pernisine, the wild-type pernisine from *A. pernix*, and proteinase K were assayed for their rates of proteolytic degradation of the normal cellular prion protein PrP^C^ and the pathological form PrP^Sc^ in bovine-brain homogenates. Proteinase K digestion is a normal step in the protocol of the commercially available Western blotting approved for testing of bovine brain for bovine spongiform encephalopathy (BSE), which was used in this study. When compared to the untreated control (Fig. 7, lane K), PrP^C^ and PrP^Sc^ from bovine-brain homogenates were degraded by both the recombinant and wild-type pernisine at 90 °C (Fig. 7a, b, lanes 6, 7, respectively), as well as by proteinase K at 37 °C (Fig. 7a, lane 8). However, the remaining level of PrP^C^ and PrP^Sc^ after treatment was related to the starting concentration of PrP^C^ and PrP^Sc^ in the samples, as demonstrated for the recombinant pernisine in Fig. 7a, b (lanes 1–6), respectively. Several different dilution ratios of PrP protein and recombinant pernisine were tested. Degradation was more effective when higher dilutions of bovine-brain homogenate were tested. Wild-type pernisine was applied in one lane only for Fig. 7a, b (both lane 7) and at a concentration that was shown to be effective for PrP^C^ and PrP^Sc^ degradation in previous studies [6]. Here, the wild-type pernisine was applied at a 1:1 (v/v) dilution of the original brain homogenate (see Concentration of brain homogenate comparable to that of recombinant protein in Fig. 7a, b, both lane 2), and it efficiently degraded PrP^C^ and PrP^Sc^ (Fig. 7a, b, lane 7, respectively). On the other hand, proteinase K completely degraded PrP^C^ (Fig. 7a, lane 8), but only partially degraded PrP^Sc^ at the same brain homogenate dilution (Fig. 7b, lane 8). These data confirmed that the wild-type and recombinant pernisine efficiently degraded both PrP^C^ and PrP^Sc^ (Fig. 7b, lanes 6 and 7), and were more efficient at degradation of PrP^Sc^ than proteinase K (Fig. 7b, lane 8).

## Discussion

Production of the thermostable protease pernisine from the *Archaeon A. pernix* has industrial potential due to its biochemical properties, such as thermostability, stability in the presence of detergents and denaturants, and ability to degrade prions [6]. However, all of these characteristics make this enzyme challenging to express in mesophilic microbial hosts, mainly due to the different thermodynamics of the environment in which its folding occurs. Here, we used the bacterium *S. rimosus* as a host for heterologous expression of recombinant pernisine, which is known for efficient protein secretion [9]. This study started with a straightforward approach of amplification of the native *pernisine* gene containing the regions that code for its native pre- and proregion (*prepropernisine*^*WT*^), along with a sequence coding for six histidines on the C-terminus, which were all placed under the relatively strong P_*tcp830*_ promoter. In this way, the constructed gene was located on the *E. coli*–*Streptomyces* replicative vector pVF, and designated as pVFPER1. A number of transformants were assayed for intracellular and extracellular activities; however, no pernisine activity was detected. SDS-PAGE, zymography and Western blotting confirmed the absence of the target enzyme.

The first measure to potentially ‘activate’ the translation of the *prepropernisine* gene was to optimise the nucleotide sequence to a more favourable codon composition for the *S. rimosus* host. Thus, the *prepropernisine*^*WT*^ gene was replaced by the codon-optimised *prepropernisine*^*CO*^ gene, which yielded plasmid construct pVFPER2. In this way, a 52-kDa protein was obtained. Zymography assays revealed a weak band at 36 kDa, which indicated that partial processing of prepropernisine^CO^ occurred; however, due to the low concentration of the processed product, it was not possible to obtain sufficient amounts for MS/MS confirmation. Additionally, Western blotting revealed additional bands at 25 ± 3 kDa, which were identified as degradation products of prepropernisine. This implies a mechanism of pernisine maturation/activation that is similar to that already observed for Tk-subtilisin [16], which is the only protease with at least 50% amino-acid identity that was investigated on protein level [18]. However, the lack of processing of propernisine to fully active pernisine here led us to consider additional strategies for heterologous expression of pernisine.

It is difficult to explain the reason why the processing of prepropernisine to the mature pernisine protein did not occur in *S. rimosus* with the codon-optimised *pernisine* gene even after the temperature pre-treatment. However, to avoid this problem we removed the preregion as well as the proregion from the codon-optimised *prepropernisine*^*CO*^ sequence, hence fusing the pernisine core structure of *prepropernisine*^*CO*^ directly to the *srT* signal sequence. This was expected to aid the export of the target protein out of the cell through the Sec secretion pathway. At the same time, however, the fusion of this native *Streptomyces* signal sequence directly to the pernisine core region of *prepropernisine*^*CO*^ might result in incorrect processing or even misfolding of the codon-optimised pernisine, as this type of protein junction does not occur in wild-type pernisine. Alternatively, if processing of the pernisine occurs intracellularly, it might be toxic to the host cell.

Therefore, with the aim to increase the success of the engineering efforts here, we constructed three different versions of codon-optimised pernisine, as described in the Results. In the first approach, the serine proteinase signal sequence *srT* from *S. rimosus* was fused to the codon-optimised *propernisine*^*CO*^, such that the recognition motif was conserved, to obtain plasmid construct pVFPER3. When the culture supernatants were assayed for propernisine in the zymography assay, the presence of the recombinant enzyme was confirmed. Although this confirmed the functionality of the *srT* signal sequence, it also showed that the processing from propernisine to mature pernisine did not occur. This might have been due to the protein folding conditions under which the recombinant propernisine was expressed, which differed significantly from the natural environment in which native pernisine is produced, in terms of temperature, concentration and ion composition [17]. Alternatively, replacement of the native signal sequence with the *srT* signal sequence might interfere with the correct folding of the recombinant enzyme. However, as it was not possible to activate the recombinant prepropernisine with the native signal sequence, we assumed that this was not the case.

Considering the results with transformants that contained pVFPER3, and based on previous studies [15], we concluded that lack of proregion cleavage was likely the main factor that affected the activation of the pernisine. As thermal activation failed, additional plasmid constructs were constructed where the proregion was completely omitted. Thus, in the second approach, we fused the promoter directly to the codon-optimised core region of *pernisine*, omitting the *srT* signal sequence to generate pVFPER4. If functional, we could expect production of pernisine intracellularly, which however, might be toxic to the cell. In a third approach, we fused the *srT* signal sequence to the codon-optimised core region of *pernisine* without the proregion (pVFPER5), this way ensuring that the A–X–A signal peptidase cleavage motif was preserved. As expected, in cultivations with transformants harbouring the pVFPER4 construct, most of the active pernisine was detected intracellularly. Interestingly, intracellular production of pernisine did not seem to have any negative effect on morphology and physiology of *S. rimosus* culture. Finally, successful production of exported, proteolytically active pernisine was confirmed in *S. rimosius* transformants containing pVFPER5. Most of the active and fully processed pernisine was detected in the culture supernatant. Interestingly, the recombinant pernisine migrated as a 23-kDa band in SDS-PAGE, although its theoretical MW was 36 kDa. MS/MS analysis of this band implied that this version of recombinant pernisine produced by *S. rimosus* is truncated at the N-terminus. This resulted in a protein of 30 kDa MW, which is still higher than the MW estimated by SDS-PAGE. This discrepancy might be due to a retained folded conformation of pernisine during the electrophoresis, which would lead to faster migration. It is known that some oligomers are resistant to SDS treatment, and this is especially common phenomenon for proteins from extremophiles [20]. On the other hand, the recombinant prepropernisine migrated at a higher MW (52 kDa) than predicted by its theoretical MW (46 kDa), as was also reported for a recombinant prepropernisine produced in *E. coli* [7]. This implied denaturation of prepropernisine under SDS-PAGE conditions, and the slower migration on SDS-PAGE might be explained by the higher number of acidic amino acids (49 amino acids) compared to the number of basic amino acids (11 amino acids) that constitute prepropernisine. Consequently, the net negative charge of prepropernisine might result in SDS repulsion, and thus slower migration during SDS-PAGE [19].

After we had confirmed the successful expression of recombinant prepropernisine and pernisine, sufficient amounts of these target proteins were obtained with the *S. rimosus* transformants containing the pVFPER3 and pVFPER5 plasmids, respectively, for further characterisation of the biochemical properties. The comparison of the CD spectra of the structural elements of prepropernisine and pernisine showed very small differences when the proregion was omitted. Small differences in the secondary structure were also seen for recombinant Tk-subtilisin [21], although the secondary structure of pernisine and its homologue Tk-subtilisin differ significantly. Of note here, Tk-subtilisin was purified in a denatured conformation and was refolded afterwards [16], whereas in the present study, pernisine was purified directly in its folded form.

The azocasein assay showed that compared to the fully processed recombinant pernisine produced by *E. coli* [7], this codon-optimised recombinant pernisine was proteolytically active at similar pH (pH 4.5–9.1) and temperature (90–110 °C), and reached its maximum proteolytic activity at the same pH and temperature, as pH 7.0 (± 0.2) and 100 (± 1)  °C, respectively. In addition, comparison between wild-type [6], recombinant fully processed pernisine from *E. coli* [7] and recombinant fully processed pernisine from *S. rimosus*, revealed that the recombinant protein from *S. rimosus* showed higher resistance to the effects of serine protease inhibitor, reducing agents, denaturants and a detergent, and thus higher residual proteolytic activities of 10% to 30%. This indicates the advantages of this recombinant pernisine from *S. rimosus* compared to pernisine produced in *E. coli*.

One of the most interesting properties of wild-type pernisine is that it degraded the infectious prion protein (PrP^Sc^) in bovine-brain homogenates from animals with BSE, and not just the normal PrP^C^ of healthy bovines. To the best of our knowledge, pernisine is one of the few enzymes that can efficiently degrade the infectious PrP^Sc^ protein [4, 6, 22, 23], with this being confirmed in the present study. When we applyed the recombinant fully processed pernisine from *S. rimosus*, we observed efficient degradation of PrP^C^ and PrP^Sc^ in the infected bovine brain homogenates. In contrast, proteinase K degraded PrP^C^ and only partially degraded PrP^Sc^. These data indicate that the recombinant pernisine retains the proteolytic activity against both PrP^C^ and PrP^Sc^ of the wild-type pernisine.

This study demonstrated that the thermostable protease pernisine that has the potential for use in a broad range of industrial applications can be produced in a *S. rimosus* host in its fully processed and active form without the need for prior activation. Considering that we demonstrated efficient secretion of the fully-active thermophilic enzyme by applying gene tools and industrial cultivation strategies that have been developed in *S. rimosus*, this host presents a valuable platform for the industrial production of pernisine and other bulk enzymes.

## Conclusions

In this study, we engineered the *prepropernisine* gene in a systematic step-by-step approach, which started with simple heterologous expression of the wild-type *prepropernisine* and *prepropernisine*^*CO*^ genes in an *S. rimosus* background. This allowed production of pernisine using the relatively straightforward approach of codon optimisation. However, the main challenge with heterologous expression of pernisine in *S. rimosus* was not the production of the pernisine protein itself, but rather the need to obtain proteolytically active pernisine through its correct processing. Therefore, the most difficult part of the engineering efforts here was to construct a chimeric protein that was composed of the *srT S. rimosus* signal sequence that was fused directly to the *prepropernisine*^*CO*^ gene from *A. pernix* after removal of the preregion and the proregion. Both versions were tested here, as *srT*-*propernisine* and *srT*-*pernisine*. Thus, the most critical point here was to construct the functional fusion between the *srT* signal sequence and the *propernisine*^*CO*^ and *pernisine*^*CO*^ genes.

Then, by applying bioinformatics analysis, a functional cleavage motif was engineered that was recognized by a wild-type *S. rimosus* signal peptidase. This was, however, only achieved with the *srT* signal sequence fused directly to the core *pernisine* gene (i.e., without the proregion). Therefore, through these protein engineering approaches, we have demonstrated the first successful production of extracellular thermostable pernisine, without the need for its subsequent pre-activation.

The biochemical analyses of the purified codon-optimised recombinant pernisine produced did not show any significant differences compared to the wild-type pernisine. Most importantly, this recombinant pernisine was also proteolytically active against the normal PrP^C^ and the infectious PrP^Sc^ in prion-infected bovine brain homogenates. The yield of this codon-optimised recombinant pernisine in *S. rimosus* achieved in this study was estimated to reach around 10 mg/L culture medium. Naturally, this system requires further industrial strain and processing improvements. However, we have demonstrated that *Streptomyces* spp. can provide potentially valuable alternatives as industrial heterologous hosts. We have also demonstrated here that application of synthetic biology approaches can be effective for the production of enzymes from thermophilic microorganisms in mesophilic industrial heterologous hosts.

## Materials and methods

### Bacterial strains and cultivation media

The plasmid constructs were prepared and propagated in *E. coli* DH10ß. Non-methylated DNA for transformation of *S. rimosus* was prepared in *E. coli* ET12567 [24]. *S. rimosus* M4018 (DSM 105900) was transformed using electroporation, as described previously [25]. *E. coli* was cultivated in 2TY medium supplemented with ampicillin (100 µg/mL), apramycin (75 µg/mL), or chloramphenicol (10 µg/mL), as required. *S. rimosus* was cultivated in tryptic soy broth that contained thiostrepton (solid medium, 30 µg/mL; liquid medium, 5 µg/mL). *S. rimosus* spores were propagated on soya-mannitol agar medium [26] supplemented with thiostrepton (30 µg/mL), at 30 °C for 8–10 days, and then stored in 20% glycerol at -80 °C. The commercially available medium 2YT was from Merck (Germany), and the minimal medium [27], tryptic soy broth [26] and seed and complex media are described in the literature [28]. All of the reagents were from Merck (Germany), if not specified otherwise.

### Sequence alignment and model structure of pernisine

The pernisine sequence (Ape263.1) was searched using BLAST, and the proteins that had > 50% amino-acid identity were further aligned using the COBALT programme [29]. Searches were performed for putative signal sequences and proregions for these proteins, using the consortium of databases known as InterPro, which includes the programmes SignaIP and Phobius [30]. Amino-acid alignment with Tk-subtilisin was performed with particular emphasis on the structural organisation (e.g., signal sequence, proregion, Ca^2+^-binding sites; see Additional file 1: Figs. S2, S4). Combining all of these data, the model structure was assembled using SWISS-MODEL [31]. The 2Z2Z and 2Z2X structures from the protein database were used as the templates for propernisine and pernisine, respectively.

### Assembly of the plasmid constructs

The pernisine gene (*Ape263.1*; 1293 bp) from the *Archaeon A. pernix* was codon-optimised using the OptimumGene algorithm (Additional file 1: Table S1, Fig. S5), and synthesised by Genscript (USA). The proregion of the engineered gene contained an NdeI restriction site at the 5′-end and a PstI restriction site at the 3′-end of the wild-type signal sequence, and a His_6_-tag and XbaI site at the 3′-end. The NdeI-*pernsyn*-XbaI fragment was introduced into the pEX-*tcp830* plasmid that had previously been digested with NdeI*/*XbaI. Furthermore, the EcoRI-*tcp830/pern*^*CO*^-EcoRI fragment was ligated into pVF, which had previously been linearised with EcoRI, to yield pVF-tcp830*/pern*^*CO*^. To produce the pVF-*tcp830*-*srT/pern*^*CO*^, the *prepropernisine*^*CO*^ gene was digested with PstI and XbaI, and the *propernisine*^*CO*^ gene, without the wild-type signal sequence, was ligated into the pEX-*tcp830*-2 vector, which already contained the *srT* signal sequence for wild-type *S. rimosus*; this vector was previously treated with PstI/XbaI. The fragment EcoRI-*tcp830*-*srT/pern*^*CO*^-EcoRI was ligated into the pVF vector that had been linearised previously with EcoRI. To remove the proregion of *propernisine*^*CO*^, the *propernisine*^*CO*^ gene was PCR amplified with primers 5′-AAAAACATATGGCCATGGCCAAGCCGC-3′ and 5′AAAAATCTAGACTAGTGGTGGTGGTGGTGGTGGG-3′, which incorporated the NdeI and XbaI restriction sites, respectively. The fragment NdeI-*pro*-*pern*^*CO*^-XbaI was ligated into the previously linearised pEX-*tcp830* plasmid with NdeI/XbaI. The fragment EcoRI-*tcp830/pro*-*pern*^*CO*^-EcoRI was further incorporated into the pVF vector via the EcoRI produced pVF-*tcp830/pro*-*pern*^*CO*^ vector. To fuse the *srT* signal sequence to the *pernisine*^*CO*^ gene after removal of the proregion, the *pernisine*^*CO*^ gene was PCR amplified using the forward primer 5′-AAAAACTGCAGCCATGGCCAAGCCGC-3′ and the reverse primer given above. The fragment PstI-*pro*-*pern*^*CO*^-XbaI was ligated into pEX-*tcp830*-2 that had been linearised with PstI/XbaI, and the pVF-*tcp830/srT*-*pro*-*pern*^*CO*^ vector was generated after ligation of the EcoRI-*tcp830/srT*-*pro*-*pern*^*CO*^-EcoRI fragment into the pVF vector. Finally, *pernisine*^*WT*^ was PCR amplified using the gDNA template of *Archaeon A. pernix* K1 and primers 5′-GCATATGGGCACCAAGATCGCCGCCATCGCCATCGCCC-3′ and 5′-GTCTAGAGTCAGTGGTGGTGGTGGTGGTGGGAGGAGACGGCGGTCTGGA-3′. The fragment NdeI-*pernisine*-XbaI was ligated into the pEX-*tcp830* plasmid treated with the same set of enzymes, and pVF-tcp830/pernisine resulted after ligation of the EcoRI-*tcp830*-*pern*-EcoRI fragment into the pVF vector (Fig. 1). The resulting plasmids were named as pVFPERx (x = 1, 2, 3, 4, 5), as illustrated in Fig. 1. The DNA sequences were verified by sequencing prior to their transformation into *S. rimosus*. All of the vectors constructed in this study are described in detail in Table 1.

### Heterologous production of pernisine using the *S. rimosus* host

The *S. rimosus* transformants were cultivated in the seed medium, and after 20 h to 24 h, 10% (v/v) inoculum was transferred into complex medium [9]. Thiostrepton (5 µg/mL) was always added to the liquid media, and the cultures were cultivated at 30 °C with constant agitation at 220 rpm for 4 days. Then, the cultures were centrifuged at 14,000×*g* for 5 min at 4 °C, and the supernatants and total cell lysates were analysed for pernisine activity. The transformants that contained the constructs with *prepropernisine*^WT^, *propernisine*^CO^ or *pernisine*^*CO*^ were tested for pernisine production. The recombinant protein was purified using affinity chromatography on Ni–NTA columns (Roche, Switzerland), according to the manufacturer instructions. Protein purities were estimated as > 90% based on SDS-PAGE analysis. Protein identities were confirmed using MS/MS. The bands at 50 kDa shown in Fig. 3a and at 23 kDa shown in Fig. 4a were cut out of the SDS-PAGE gels, and the samples were analysed for their C-termini and N-termini at the European Molecular Biology Laboratory (Germany), as described previously [7].

### Protease activity

Protein concentrations were determined either spectrophotometrically (pernisine, $\varepsilon_{{280{\text{nm}}}}^{1\% }$ = 60,850/M/cm; proteolytically active pernisine, $\varepsilon_{{280{\text{nm}}}}^{1\% }$ = 57,870/M/cm) or by the Bradford method using protein assays (BioRad, USA) with bovine serum albumin as the standard, as described previously [6]. For qualitative determination of the proteolytic activity of the recombinant pernisine, zymography procedures were used with standard SDS-PAGE, as described below. To characterise the recombinant pernisine, azocasein assays were used, as described previously [6]. The samples were assayed in triplicate. Data are expressed as means ± standard errors. The relative protease activities of pernisine under the different conditions were expressed as described in Additional file 1: Materials and Methods.

### SDS-PAGE/zymography, Western blotting and dot blots

Protein samples (10 µg) were analysed using SDS-PAGE with 15% polyacrylamide gels, and visualised using SimplyBlue Safe staining (ThermoFischer Scientific, USA). Zymogram gels were prepared using the same procedures as for the SDS-PAGE gels, with the addition of 0.1% casein, and compared to the SDS-PAGE gels, one tenth of the sample size was loaded onto the gels. To determine the qualitative proteolytic activity of the recombinant pernisine, zymography was carried out with standard SDS-PAGE procedures, as described previously [6], with the details described in Additional file 1: Supporting Materials and Methods.

### Circular dichroism spectroscopy

The CD spectra of pernisine (0.2 mg/mL) were recorded with a spectrometer (J-1500; Jasco, Japan) at 25 °C and pH 8.0. For experimental purposes, 10 mM Tris–HCl buffer containing 1 mM CaCl_2_ was used. The CD spectra of pernisine were measured at the far-UV wavelengths from of 200 nm to 250 nm, every 1 nm, in a cuvette with a 1-mm path length. The secondary structures (e.g., α-helices, β-sheets, unordered structures) were estimated using the CONTIN [32] programme and the Sreenan and Woody protein reference datasets. Prior to the software analysis, the baselines were subtracted from the CD spectra and smoothed using Savitsky-Golay filtering analysis. The mean amino-acid ellipticity was calculated using a mean amino-acid mass of 102 Da.

### Bovine-brain homogenate and degradation of prion proteins

Degradation of the PrP^C^ and infectious PrP^Sc^ prion proteins in bovine-brain homogenates was monitored in vitro using Prionics-Check Western kits (Prionics AG, Thermo Fischer Scientific, USA). The detailed procedure is described in Additional file 1: Materials and Methods.

## Supplementary information

**Additional file 1: Table S1.** Codon distribution of the wild-type pernisine sequence from *A. pernix* and the codon-optimised pernisine sequence from *S. rimosus*. **Figure S1.** Tandem mass spectrometry (MS/MS) analysis of the N-termini and C-termini of the codon-optimised prepropernisine (A) and the codon-optimised processed pernisine (B). Individual identified peptides are aligned with the pernisine sequence. **Figure S2.** Model of the three-dimensional structure of propernisine (A) and processed pernisine (B), with the Ca^2+^ binding sites indicated (C). Note that the first 31 amino acids are not shown in the model structure of pernisine. Propernisine consists of the proregion (V31-A92; turquois), the catalytic triad (D149, H184, S355), and the Ca^2+^ ions (green spheres). Processed pernisine form (Q93-V430). Frame C presents four selected Ca^2+^ binding sites that interact with either amino acids or water (H_2_O). **Figure S3.** Production of codon-optimised srT-pernisine (from plasmid construct pVFPER5) in total cell lysates from *S. rimosus* grown in different media. TSB, tryptic soy broth; MM, minimal medium; G3, G4, G5, complex production media at 3, 4 and 5 days. The protein was transferred onto nitrocellulose membranes as dot blots, and His_6_-tagged pernisine was detected using anti-His_5_ antibodies. **Figure S4.** Amino-acid alignment of pernisine and Tk-subtilisin with the Ca^2+^ binding sites indicated. Yellow, identical amino acids; green, related amino acids. Known Ca^2+^ binding sites from Tk-subtilisin are marked with coloured asterisks. Site 1, blue; site 2, red; site 3, black; site 4, green; site 5, violet; site 6, orange; site 7, grey. **Figure S5.** Comparison of the nucleotide sequences of *prepropernisine*^*WT*^ and *propernisine*^*CO*^. The predicted amino-acid sequence of the prepropernisine is indicated in the single-letter amino-acid code. Differences in the DNA sequences are shown in bold letters. Single underlined, amino acids of the signal sequence; double underlined, amino acids of the predicted proregion; remaining amino acids, active pernisine. The amino acids involved in the predicted catalytic triad are in yellow, as Asp149, His184 and Ser355. The start codon is marked with an asterisk (*); the stop codon with a hyphen (−). Note that only ATG was used as the start codon for the heterologous expression.

## Data Availability

All data generated or analysed during this study are included in this published article and its additional files.
