# Supplementary material for: Extracellular production of the engineered thermostable protease pernisine from Aeropyrum pernix K1 in Streptomyces rimosus
Source: Microb Cell Fact. 2019 Nov 7;18:196. doi: 10.1186/s12934-019-1245-3 (PMC6839199; doi:10.1186/s12934-019-1245-3)
Supplement: Supplementary file 1 — Additional file 1: Table S1. Codon distribution of the wild-type pernisine sequence from A. pernix and the codon-optimised pernisine sequence from S. rimosus. Figure S1. Tandem mass spectrometry (MS/MS) analysis of the N-termini and C-termini of the codon-optimised prepropernisine (A) and the codon-optimised processed pernisine (B). Individual identified peptides are aligned with the pernisine sequence. Figure S2. Model of the three-dimensional structure of propernisine (A) and processed pernisine (B), with the Ca2+ binding sites indicated (C). Note that the first 31 amino acids are not shown in the model structure of pernisine. Propernisine consists of the proregion (V31-A92; turquois), the catalytic triad (D149, H184, S355), and the Ca2+ ions (green spheres). Processed pernisine form (Q93-V430). Frame C presents four selected Ca2+ binding sites that interact with either amino acids or water (H2O). Figure S3. Production of codon-optimised srT-pernisine (from plasmid construct pVFPER5) in total cell lysates from S. rimosus grown in different media. TSB, tryptic soy broth; MM, minimal medium; G3, G4, G5, complex production media at 3, 4 and 5 days. The protein was transferred onto nitrocellulose membranes as dot blots, and His6-tagged pernisine was detected using anti-His5 antibodies. Figure S4. Amino-acid alignment of pernisine and Tk-subtilisin with the Ca2+ binding sites indicated. Yellow, identical amino acids; green, related amino acids. Known Ca2+ binding sites from Tk-subtilisin are marked with coloured asterisks. Site 1, blue; site 2, red; site 3, black; site 4, green; site 5, violet; site 6, orange; site 7, grey. Figure S5. Comparison of the nucleotide sequences of prepropernisineWT and propernisineCO. The predicted amino-acid sequence of the prepropernisine is indicated in the single-letter amino-acid code. Differences in the DNA sequences are shown in bold letters. Single underlined, amino acids of the signal sequence; double underlined, amino acids of the [file 12934_2019_1245_MOESM1_ESM.docx]

**Additional file 1**

**Extracellular production of the engineered thermostable protease pernisine from *Aeropyrum pernix* K1 in *Streptomyces rimosus***

Marko Šnajder^1,¥^, Andres Felipe Carrillo Rincon^1^, Vasilka Magdevska^1,3+^, Miha Bahun^1^, Luka Kranjc^1^, Maja Paš^1^, Polona Juntes^4^, Hrvoje Petković^1^*, Nataša Poklar Ulrih^1,2^*

Table S1

Figures S1-S5

Additional Material and Methods

Additional Results

Additional References

**Table S1.** Codon distribution of the wild-type pernisine sequence from *A. pernix* and the codon-optimised pernisine sequence from *S. rimosus*.

| **Codon** | **Amino** | **Fraction^†^** | **Pernisine** | |  | **Codon** | **Amino** | **Fraction^†^** | **Pernisine** | |
| --- | --- | --- | --- | --- | --- | --- | --- | --- | --- | --- |
|  | **acid** |  | **Wild-type** | **Codon optimised** |  |  | **acid** |  | **Wild-type** | **Codon optimised** |
| TAA | ‡ | 0.00 | 0 | 0 |  | TTA | L | 0.00 | 0 | 0 |
| TAG | ‡ | 0.14 | 0 | 0 |  | TTG | L | 0.02 | 1 | 0 |
| TGA | ‡ | 0.86 | 1 | 1 |  | CTT | L | 0.01 | 5 | 0 |
| GCT | A | 0.02 | 22 | 1 |  | CTC | L | 0.24 | 6 | 1 |
| GCC | A | 0.52 | 14 | 52 |  | CTA | L | 0.00 | 6 | 0 |
| GCA | A | 0.03 | 6 | 1 |  | CTG | L | 0.73 | 9 | 26 |
| GCG | A | 0.44 | 12 | 0 |  | AAA | K | 0.06 | 2 | 0 |
| CGT | R | 0.06 | 0 | 0 |  | AAG | K | 0.94 | 10 | 12 |
| CGC | R | 0.44 | 0 | 5 |  | ATG | M | 1.00 | 4 | 4 |
| CGA | R | 0.02 | 0 | 0 |  | TTT | F | 0.03 | 1 | 0 |
| CGG | R | 0.43 | 0 | 0 |  | TTC | F | 0.97 | 2 | 3 |
| AGA | R | 0.00 | 2 | 0 |  | CCT | P | 0.03 | 14 | 0 |
| AGG | R | 0.04 | 3 | 0 |  | CCC | P | 0.34 | 5 | 7 |
| AAT | N | 0.01 | 3 | 0 |  | CCA | P | 0.02 | 9 | 0 |
| AAC | N | 0.99 | 14 | 17 |  | CCG | P | 0.61 | 4 | 25 |
| GAT | D | 0.08 | 13 | 0 |  | TCT | S | 0.01 | 3 | 0 |
| GAC | D | 0.92 | 25 | 38 |  | TCC | S | 0.39 | 4 | 30 |
| TGT | C | 0.12 | 0 | 0 |  | TCA | S | 0.02 | 3 | 0 |
| TGC | C | 0.88 | 0 | 0 |  | TCG | S | 0.27 | 1 | 0 |
| CAA | Q | 0.03 | 0 | 0 |  | AGT | S | 0.02 | 1 | 0 |
| CAG | Q | 0.97 | 6 | 6 |  | AGC | S | 0.28 | 18 | 0 |
| GAA | E | 0.15 | 1 | 0 |  | ACT | T | 0.01 | 13 | 0 |
| GAG | E | 0.85 | 16 | 17 |  | ACC | T | 0.66 | 4 | 21 |
| GGT | G | 0.08 | 10 | 0 |  | ACA | T | 0.02 | 1 | 0 |
| GGC | G | 0.63 | 20 | 52 |  | ACG | T | 0.31 | 3 | 0 |
| GGA | G | 0.05 | 9 | 0 |  | TGG | W | 1.00 | 7 | 7 |
| GGG | G | 0.24 | 13 | 0 |  | TAT | Y | 0.08 | 6 | 0 |
| CAT | H | 0.08 | 2 | 0 |  | TAC | Y | 0.92 | 9 | 15 |
| CAC | H | 0.92 | 5 | 7 |  | GTT | V | 0.02 | 17 | 0 |
| ATT | I | 0.02 | 8 | 0 |  | GTC | V | 0.43 | 10 | 42 |
| ATC | I | 0.94 | 8 | 35 |  | GTA | V | 0.06 | 10 | 0 |
| ATA | I | 0.03 | 19 | 0 |  | GTG | V | 0.48 | 11 | 6 |

**^†^**Fraction of relative use of each codon in its synonymous codon family in *S. rimosus*

‡ stop codon

**A**

**
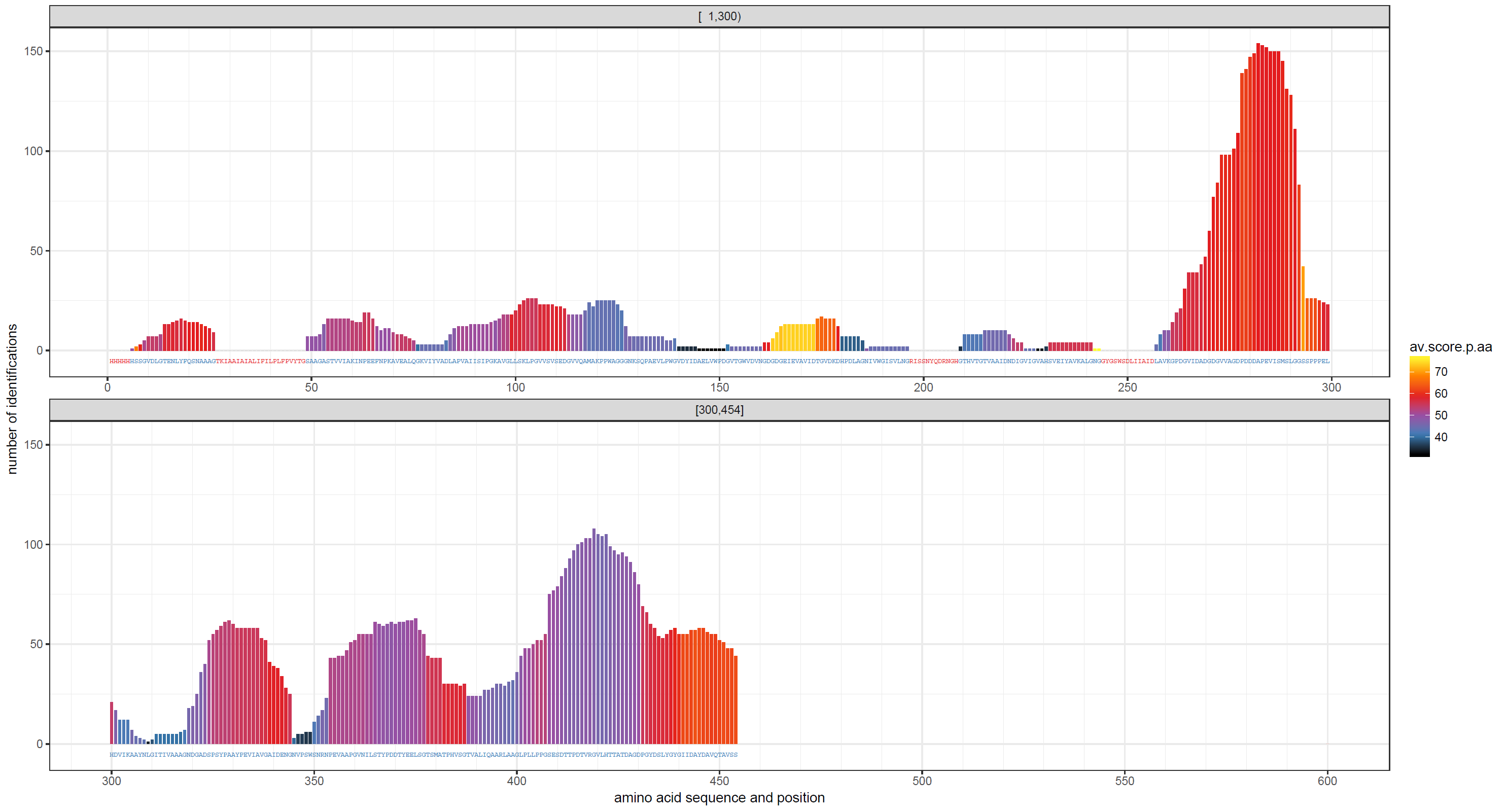
**

**B**

**
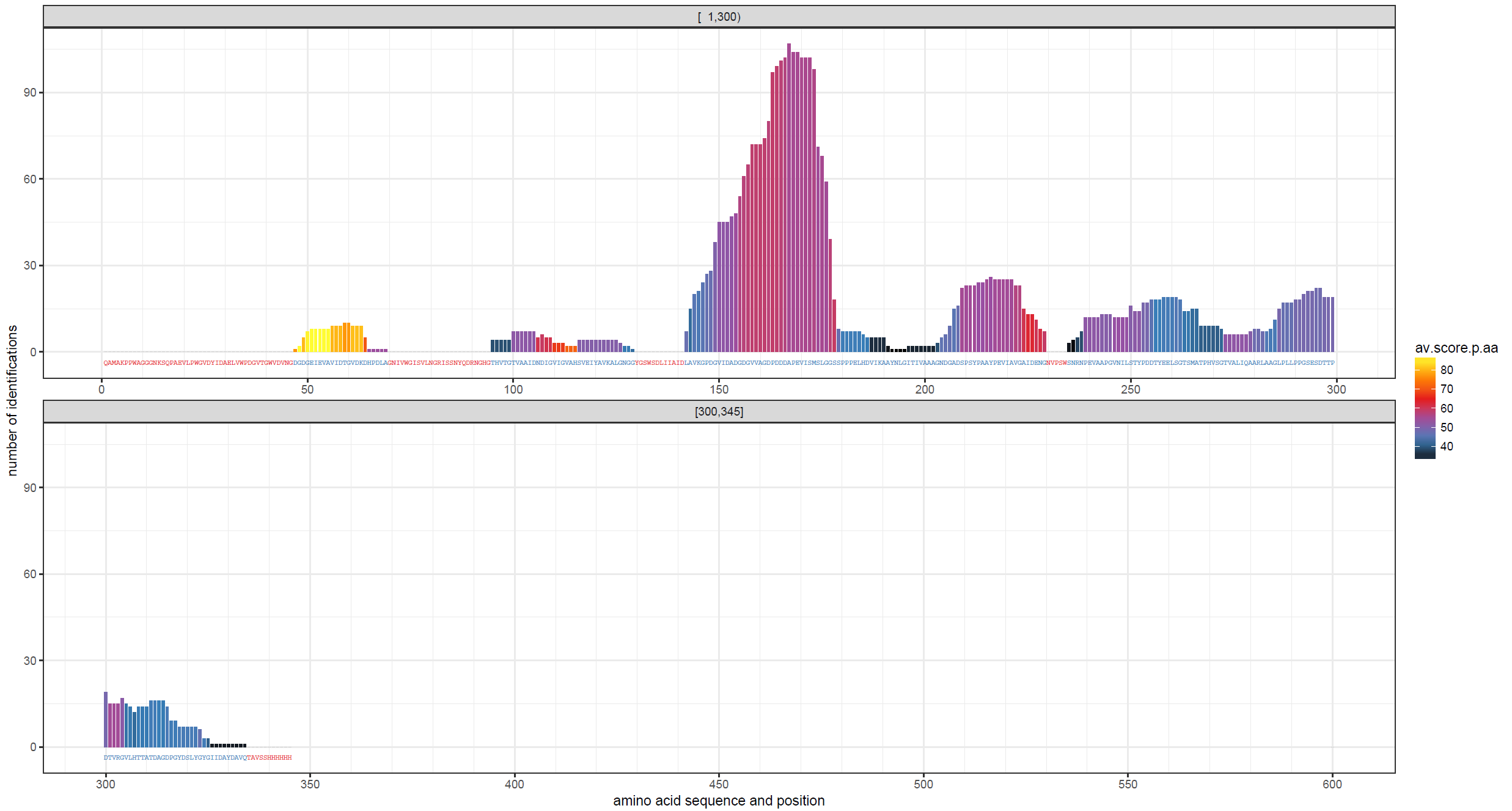
**

**Figure S1.** Tandem mass spectrometry (MS/MS) analysis of the N-termini and C-termini of the codon-optimised prepropernisine (A) and the codon-optimised processed pernisine (B). Individual identified peptides are aligned with the pernisine sequence.


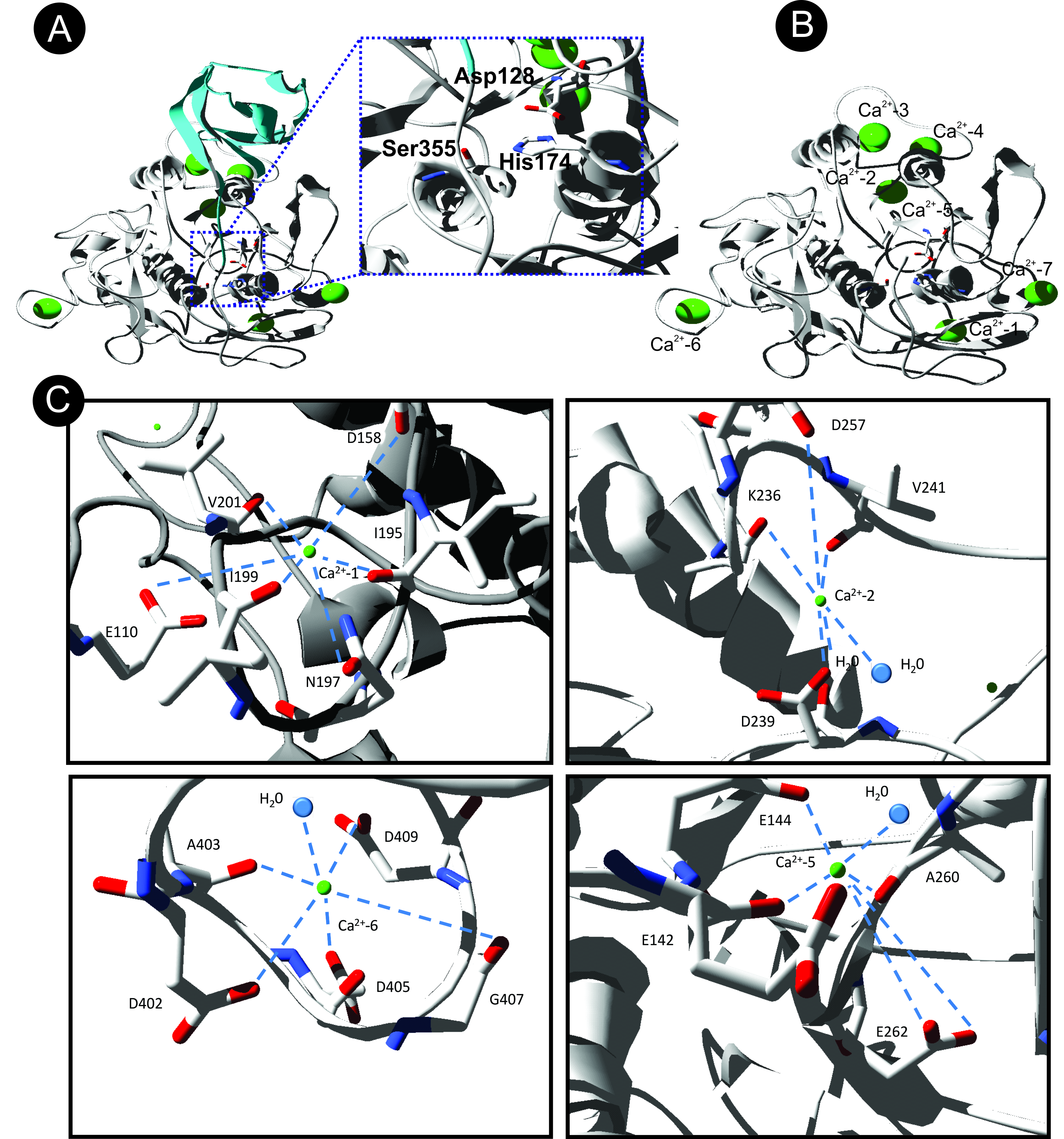


**Figure S2.** Model of the three-dimensional structure of propernisine (A) and processed pernisine (B), with the Ca^2+^ binding sites indicated (C). Note that the first 31 amino acids are not shown in the model structure of pernisine. Propernisine consists of the proregion (V31-A92; turquois), the catalytic triad (D149, H184, S355), and the Ca^2+^ ions (green spheres). Processed pernisine form (Q93-V430). Frame C presents four selected Ca^2+^ binding sites that interact with either amino acids or water (H_2_O).


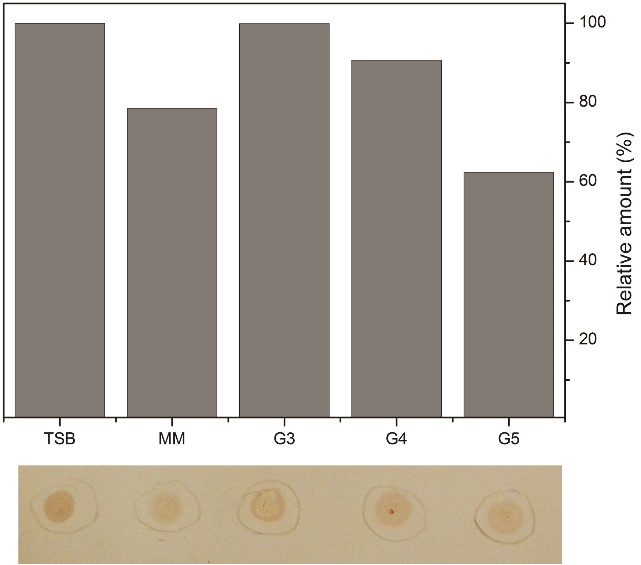


**Figure S3.** Production of codon-optimised srT-pernisine (from plasmid construct pVFPER5) in total cell lysates from *S. rimosus* grown in different media. TSB, tryptic soy broth; MM, minimal medium; G3, G4, G5, complex production medium at 3, 4 and 5 days. The protein was transferred onto nitrocellulose membranes as dot blots, and His_6_-tagged pernisine was detected using anti-His_5_ antibodies.


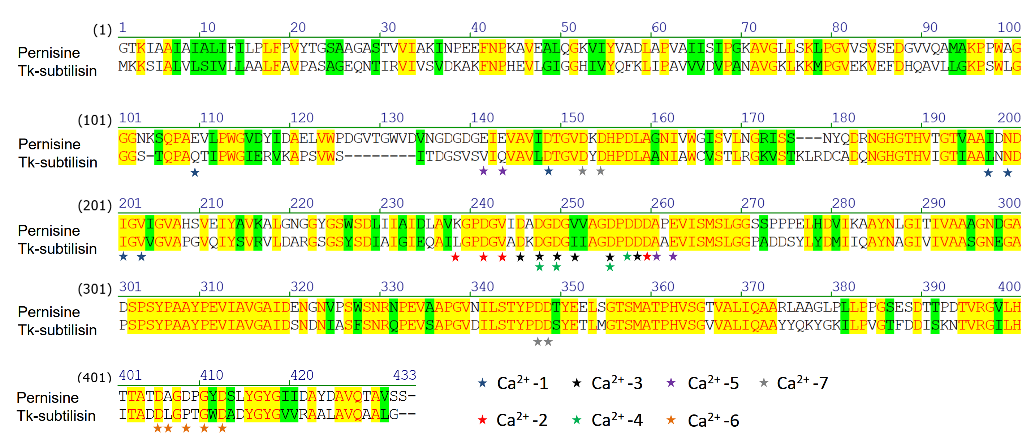


**Figure S4.** Amino-acid alignment of pernisine and Tk-subtilisin with the Ca^2+^ binding sites indicated. Yellow, identical amino acids; green, related amino acids. Known Ca^2+^ binding sites from Tk-subtilisin are marked with coloured asterisks. Site 1, blue; site 2, red; site 3, black; site 4, green; site 5, violet; site 6, orange; site 7, grey.

1 100

wtPernisine **G**TGGG**A**AC**T**AA**G**ATCGC**G**GCTAT**T**GC**G**ATCGC**G**CTGATCTT**C**AT**T**CTGCC**T**CT**C**TTCCC**T**GT**T**TA**T**ACGGG**A**TC**G**GC**G**GCTGG**G**GCTAGCAC**G**GT**T**GT**G**A

coPernisine ATGGGCACCAAGATCGCCGCCATCGCCATCGCCCTGATCTTCATCCTGCCGCTGTTCCCGGTCTACACCGGCTCCGCTGCAGGCGCCTCCACCGTCGTCA

* G T K I A A I A I A L I F I L P L F P V Y T G S A A G A S T V V

101 200

wtPernisine T**A**GC**T**AA**G**AT**T**AA**T**CC**T**GA**G**GA**G**TTTAA**C**CC**T**AA**G**GC**G**GTGGA**G**GC**T**CT**T**CA**G**GGCAA**G**GT**A**AT**A**TATGTTGCTGATCTGGC**C**CC**C**GT**T**GC**T**AT**A**AT**T**AG

coPernisine TCGCCAAGATCAACCCCGAGGAGTTCAACCCGAAGGCCGTCGAGGCCCTCCAGGGCAAGGTCATCTACGTCGCCGACCTGGCCCCGGTCGCCATCATCTC

I A K I N P E E F N P K A V E A L Q G K V I Y V A D L A P V A I I S

201 300

wtPernisine CAT**A**CC**A**GG**A**AA**G**GC**T**GT**A**GG**C**CTGCT**C**TCTAAACT**A**CC**T**GG**T**GT**T**GT**CAG**CGTTTC**C**GA**G**GA**C**GG**C**GT**G**GTCCAGGCTATGGCCAA**G**CCGCC**G**TGGGC**T**

coPernisine CATCCCGGGCAAGGCCGTCGGCCTGCTGTCCAAGCTGCCGGGCGTCGTCTCCGTCTCCGAGGACGGCGTCGTCCAGGCCATGGCCAAGCCGCCGTGGGCC

I P G K A V G L L S K L P G V V S V S E D G V V Q A M A K P P W A

301 400

wtPernisine GGCGG**C**GG**G**AA**T**AA**G**TCTCA**G**CC**T**GC**C**GA**G**GT**C**CTGCC**T**TGGGG**T**GT**C**GA**C**TATAT**C**GA**T**GC**C**GA**G**CT**A**GT**A**TGGCC**C**GATGG**G**GT**T**ACCGG**C**TGGGTTG

coPernisine GGCGGCGGCAACAAGTCCCAGCCGGCCGAGGTCCTGCCGTGGGGCGTCGACTACATCGACGCCGAGCTGGTCTGGCCGGACGGCGTCACCGGCTGGGTCG

G G G N K S Q P A E V L P W G V D Y I D A E L V W P D G V T G W V

401 500

wtPernisine ACGT**T**AA**C**GGTGACGG**G**GA**C**GG**C**GA**G**AT**A**GA**G**GTTGCCGT**T**AT**T**GA**C**AC**T**GGTGT**C**GA**T**AA**G**GA**C**CATCC**C**GACCT**T**GCAGGCAACATTGT**C**TGGGG**G**AT

coPernisine ACGTCAACGGCGACGGCGACGGCGAGATCGAGGTCGCCGTCATCGACACCGGCGTCGACAAGGACCACCCGGACCTGGCCGGCAACATCGTCTGGGGCAT

D V N G D G D G E I E V A V I D T G V D K D H P D L A G N I V W G I

501 600

wtPernisine **ATC**TGT**TT**TGAA**C**GGC**A**G**G**AT**ATC**CTC**C**AACTACCA**G**GAT**A**G**A**AA**C**GGCCA**C**GGTAC**A**CA**C**GT**A**AC**G**GG**C**AC**T**GT**A**GCCGC**C**AT**A**GACAACGATAT**A**GG**G**

coPernisine CTCCGTCCTGAACGGCCGCATCTCCTCCAACTACCAGGACCGCAACGGCCACGGCACCCACGTCACCGGCACCGTCGCCGCCATCGACAACGACATCGGC

S V L N G R I S S N Y Q D R N G H G T H V T G T V A A I D N D I G

601 700

wtPernisine GT**G**AT**A**GG**G**GT**T**GC**A**CACAGCGTGGA**G**ATCTA**C**GC**C**GTTAAAGCTCT**C**GG**T**AA**C**GG**G**GG**T**TACGG**C**AGCTGG**AGC**GA**C**CTTAT**A**AT**A**GC**T**AT**A**GACCT**T**G

coPernisine GTCATCGGCGTCGCCCACTCCGTCGAGATCTACGCCGTCAAGGCCCTGGGCAACGGCGGCTACGGCTCCTGGTCCGACCTGATCATCGCCATCGACCTGG

V I G V A H S V E I Y A V K A L G N G G Y G S W S D L I I A I D L

701 800

wtPernisine C**T**GTGAA**G**GG**G**CCGGA**C**GG**C**GT**A**AT**T**GACGC**T**GATGG**A**GA**T**GG**C**GT**C**GT**C**GCTGG**G**GATCC**A**GA**C**GA**T**GATGC**T**CC**A**GA**G**GT**T**AT**CTCC**ATGAGCCT**A**GG

coPernisine CCGTCAAGGGCCCCGACGGCGTGATCGACGCCGACGGCGACGGCGTCGTGGCCGGCGACCCGGACGACGACGCCCCCGAGGTCATCTCCATGTCCCTGGG

A V K G P D G V I D A D G D G V V A G D P D D D A P E V I S M S L G

801 900

wtPernisine **T**GGGAGCAGCCC**A**CC**A**CC**A**GAACT**C**CA**C**GA**C**GT**T**ATCAA**G**GC**G**GC**G**TA**C**AACCT**T**GG**A**AT**A**AC**T**AT**T**GT**C**GC**A**GC**A**GC**G**GG**T**AA**C**GA**C**GG**G**GC**G**GAC**AGC**

coPernisine CGGCTCCTCCCCGCCGCCCGAGCTGCACGACGTCATCAAGGCCGCCTACAACCTGGGCATCACCATCGTCGCCGCCGCCGGCAACGACGGCGCCGACTCC

G S S P P P E L H D V I K A A Y N L G I T I V A A A G N D G A D S

901 1000

wtPernisine CC**C**TC**A**TA**C**CC**T**GC**A**GC**C**TACCC**T**GA**G**GT**A**AT**A**GCGGT**A**GGCGC**T**AT**A**GA**C**GA**G**AACGG**C**AA**C**GT**A**CC**T**AGCTGG**AGC**AA**TA**G**A**AA**C**CC**T**GA**G**GT**T**GC**T**G

coPernisine CCGTCCTACCCGGCCGCCTACCCCGAGGTGATCGCCGTGGGCGCCATCGACGAGAACGGCAACGTCCCGTCCTGGTCCAACCGCAACCCCGAGGTCGCCG

P S Y P A A Y P E V I A V G A I D E N G N V P S W S N R N P E V A

1001 1100

wtPernisine CACC**T**GG**A**GT**G**AACAT**A**CT**A**AG**C**ACCTA**C**CC**C**GACGA**T**AC**C**TA**T**GA**G**GA**G**CTG**AGC**GGCAC**TAGC**ATGGC**G**AC**T**CC**A**CA**C**GT**G**TC**A**GG**G**AC**T**GT**G**GC**T**CT

coPernisine CCCCGGGCGTCAACATCCTGTCCACCTACCCGGACGACACCTACGAGGAGCTGTCCGGCACCTCCATGGCCACCCCGCACGTGTCCGGCACCGTGGCCCT

A P G V N I L S T Y P D D T Y E E L S G T S M A T P H V S G T V A L

1101 1200

wtPernisine **A**AT**A**CAGGCTGCC**A**G**G**CTGGCCGC**T**GGCCT**C**CC**T**CT**A**CT**C**CC**T**CCGGG**A**AG**C**GA**GAGT**GA**C**AC**T**AC**T**CC**A**GACACCGTG**A**GGGG**C**GT**A**CTGCATAC**T**AC**T**

coPernisine GATCCAGGCCGCCCGCCTGGCCGCCGGCCTGCCGCTGCTGCCGCCGGGCTCCGAGTCCGACACCACCCCGGACACCGTCCGCGGCGTCCTGCACACCACC

I Q A A R L A A G L P L L P P G S E S D T T P D T V R G V L H T T

1201 1293

wtPernisine GC**T**AC**T**GA**C**GC**G**GG**A**GACCC**A**GG**C**TACGATAGCCTGTATGG**A**TACGG**T**AT**C**AT**A**GACGCCTA**T**GACGC**C**GT**G**CA**G**AC**T**GC**C**GTCTC**A**AG**C**TGA

coPernisine GCCACCGACGCCGGCGACCCCGGCTACGACTCCCTGTACGGCTACGGCATCATCGACGCCTACGACGCCGTCCAGACCGCCGTCTCCTCCTGA

A T D A G D P G Y D S L Y G Y G I I D A Y D A V Q T A V S S -

**Figure S5.** Comparison of the nucleotide sequences of *prepropernisine^WT^* and *prepropernisine^CO^*. The predicted amino-acid sequence of the prepropernisine is indicated in the single-letter amino-acid code. Differences in the DNA sequences are shown in bold letters. Single underlined, amino acids of the signal sequence; double underlined, amino acids of the predicted proregion; remaining amino acids, active pernisine. The amino acids involved in the predicted catalytic triad are in yellow, as Asp149, His184 and Ser355. The start codon is marked with an asterisk (*); the stop codon with a hyphen (–). Note that only ATG was used as the start codon for the heterologous expression.

**Additional Materials and Methods**

**Protease activity**

The relative activities were expressed as percentages, as the activities under the specified conditions divided by the maximal activity obtained, as given in Equation (S1):

$a\left( relative \right)=\frac{a_{X}}{a_{max}}\times100\left[ \% \right]$ (S1).

Initially, the pernisine optimum activities were defined with the standard azocasein assays. These were carried out at different temperatures (40-120 °C) and pHs (2.0-12.0). The buffers used contained 1 mM CaCl_2_, and were based on: pH 2.0 to 4.0, 50 mM glycine-HCl; pH 6.0 to 8.0, 50 mM HEPES; and pH 9.0 to 12.0, 50 mM glycine-NaOH. The pH at each incubation temperature was calculated according to the (dpH/dT)_P_ correction coefficient [1]. Contour graphs of temperature and pH dependence against pernisine relative activity were constructed using the Origin programme. Similarly, the thermostability of pernisine was evaluated using azocasein assays at different temperatures (40, 80, 98 °C) and incubation times (0, 10, 60, 120, 180, 240 min) in 50 mM Tris-HCl, pH 8.0, with 1 mM CaCl_2_.

To evaluate the effects of the inhibitors, reductants, denaturants and a detergent on the pernisine proteolytic activity, the samples in the reaction mixtures were incubated at room temperature for 10 min prior to the azocasein assays. The residual activities are expressed according to Equation (S2):

$a\left( residual \right)=\frac{a_{0}-a}{a_{0}}\times100\left[ \% \right]$ (S2).

**SDS-PAGE/ zymography, Western blotting, and dot blots**

Cell lysates were normalised according to cell density. Cells were lysed by sonication (Sonics, USA) at amplitude 40% (stepped microtips) for 5 min on ice. For Western blotting, the protein was electrotransferred from the gels to PVDF membranes. The membranes were blocked for 1 h at room temperature in 5% (w/v) non-fat dried milk in Tris-buffered saline with 0.05% (v/v) Tween 20 (TBST). His_6_-tagged pernisine was detected using rabbit polyclonal anti-histidine antibodies (Abcam, UK; 1:1,000 dilution), with incubation at room temperature for 1 h. The bound antibodies were detected with horseradish-peroxidase-conjugated goat anti-rabbit IgG antibodies (Jackson ImmunoResearch, UK; 1:2,000 dilution). Visualisation was performed using the ELC detection reagent, according to the manufacturer instructions (GE Healthcare, USA). The dot blot analysis was carried out using anti-His_5_ antibodies (Qiagen, Germany; 1:1,000 dilution), according to the manufacturer instructions. Each dot was quantified using the ImageJ software [2], and represents a different time of pernisine production.

**Bovine-brain homogenate and degradation of prion proteins**

Degradation of the PrP^C^ and infectious PrP^Sc^ prion proteins in bovine-brain homogenates was monitored *in vitro* using Prionics-Check Western kits (Prionics AG, Thermo Fischer Scientific, USA). The bovine brain used to monitor degradation of PrP^c^ was collected from healthy slaughtered cattle, and the bovine brain used to monitor degradation of the infectious PrP^Sc^ was collected from cattle diagnosed with bovine spongiform encephalopathy. The brain homogenates were prepared with the working dilution of the homogenisation buffer from the kits, homogenised with a homogeniser (FASTH; ConsulTH, Italy) and homogenisation containers (Prypcone; ConsulTH, Italy). The homogenates were diluted further with ultraclean water. The reaction mixtures were prepared with the cell or bovine spongiform encephalopathy brain homogenates. After homogenisation and preparation of the reaction mixtures with the test enzymes, the original protocol of the kits was followed. The reaction mixtures contained: 5 µL wild type (1.04 µg) or recombinant (0.44 µg) pernisine, 3 µL 1-fold to 5-fold diluted bovine-brain homogenate, and TBS buffer containing 1.5 mM CaCl_2_, to the total volume of 15 µL. The proteolytic activities of the pernisines were 8 U per reaction (Šnajder et al., 2012). The kit control samples (‘Ready to use’) and proteinase K from the kits were used as detailed in the kit instructions. The proteins from the 15-µL reaction mixtures were separated on precast NuPAGE gels (12% Bis-Tris; Invitrogen), and analysed by Western blotting, using the 6H4 primary monoclonal anti-PrP antibodies (working dilution, 1:5,000), with the secondary antibody labelled with alkaline phosphatase (working dilution, 1:5,000), from the kits. Visualisation was with CDP-Star in luminiscence buffer, with the membrane exposed to an X-ray film, as detailed in the kit instructions. To determine the effects of bovine-brain homogenate concentration, the bovine brain homogenates were diluted by up to five-fold, with no significant changes seen for the Western blotting signals obtained.

**Additional Results**

**Engineering of the pernisine core region of *prepropernisine^CO^* fused directly to the signal sequence *srT***

Based on the SignalP 3.0 software, the signal sequence of wild-type prepropernisine was identified, which encompassed the first 24 amino acids, with the cleavage site motif at G^24^-S^25^, as described previously [3]. However, the alternative Pred-TAT software proposed the signal sequence cleavage site as A^27^-G^28^. These contrasting results lead us to perform homology alignment with pernisine homologues from other microorganisms. Amino-acid sequence alignment of prepropernisine was performed with non-redundant protein sequences that shared at least 50% amino-acid identity. Here, 17 prepropernisine homologues were identified. However, **only Tk-subtilisin had been investigated previously at the protein level** [4]. **Based on all of these data, including the protein alignment with Tk-subtilisin, the** cleavage site A^27^-G^28^ was used (Fig. 1). To investigate further the effects that the proregion had on pernisine activity, homologous domains were searched for using the InterPro consortium of bioinformatic databases, as described in the Methods. The InterPro software identified the peptidase S8 propeptide domain in propernisine as the amino acids from G^28^ to M^94^, which was similar to the proregion based on the alignment with Tk-subtilisin, which additionally supported our previous findings [3]. The chosen proregion in the protein sequence from G^28^ to Q^92^ was also represented in the three-dimensional model structure of propernisine (Additional file 1: Fig. S2). In this model, the first 92 amino acids form a structure that blocks access of the substrate to the active site, which is represented by the catalytic triad of D149, H184 and S355. Thus, this predicted proregion was in agreement with the Tk-subtilisin structure, and was used in the present study for the design of the pernisine constructs (with *propernisine^CO^* and *pernisine^CO^*) without the proregion.

**Selection of production medium**

To increase the yield, different media were evaluated for cultivation of these *S. rimosus* transformants for production of pernisine and srT-pernisine, both of which were expressed under the *tcp830* promoter (Fig. 5). Similar strategies have been described for *S. lividans* [5, 6]. Cultivation was carried out in minimal medium and tryptic soy broth, which has often been used for cultivation of *Streptomyces* spp. [7]. In addition, the complex seed medium and complex medium used for oxytetracycline production were evaluated, as described previously [8]. After testing of all four of these media, and based on dot-blot and zymography assays, the highest production of recombinant pernisine was obtained in complex medium (Additional file 1: Fig. S3). Therefore, all of the further experiments were carried out in complex medium. The highest signal was observed with complex medium at 3 days after inoculation (Fig. 5D). Tryptic soy broth showed similar results to complex medium, while the minimal medium resulted in significantly lower intensity compared to the highest production in complex medium (Additional file 1: Fig. S3).

**Additional references**

1. Beynon RJ, Easterby JS: *Buffer solutions.* Oxford: IRL Press at Oxford University Press; 1996.

2. Schneider CA, Rasband WS, Eliceiri KW: NIH Image to ImageJ: 25 years of image analysis. *Nat Met* 2012, 9:671.

3. Šnajder M, Vilfan T, Černilec M, Rupreht R, Popović M, Juntes P, Šerbec VČ, Ulrih NP: enzymatic degradation of PrP^Sc^ by a protease secreted from *Aeropyrum pernix* K1. *PLoS ONE* 2012, 7:e39548.

4. Tanaka S, Saito K, Chon H, Matsumura H, Koga Y, Takano K, Kanaya S: Crystal structure of unautoprocessed precursor of subtilisin from a hyperthermophilic archaeon: evidence for Ca^2+^-induced folding. *J Biol Chem* 2007, 282:8246-8255.

5. Hamed MB, Karamanou S, Ólafsdottir S, Basílio JSM, Simoens K, Tsolis KC, Van Mellaert L, Guðmundsdóttir EE, Hreggvidsson GO, Anné J, et al: Large-scale production of a thermostable *Rhodothermus marinus* cellulase by heterologous secretion from *Streptomyces lividans*. *Microb Cell Fact* 2017, 16:232.

6. Sevillano L, Vijgenboom E, van Wezel GP, Díaz M, Santamaría RI: New approaches to achieve high level enzyme production in *Streptomyces lividans*. *Microb Cell Fact* 2016, 15:28.

7. Tanaka S-i, Matsumura H, Koga Y, Takano K, Kanaya S: Identification of the interactions critical for propeptide-catalyzed folding of Tk-subtilisin. *J Mol Biol* 2009, 394:306-319.

8. Carrillo Rincón AF, Magdevska V, Kranjc L, Fujs Š, Müller R, Petković H: Production of extracellular heterologous proteins in *Streptomyces rimosus*, producer of the antibiotic oxytetracycline. *Appl Microbiol Biot* 2018, 102:2607–20.
